# Alteration in synaptic nanoscale organization dictates amyloidogenic processing in Alzheimer's disease

**DOI:** 10.1016/j.isci.2020.101924

**Published:** 2020-12-11

**Authors:** Shekhar Kedia, Pratyush Ramakrishna, Pallavi Rao Netrakanti, Nivedita Singh, Sangram S. Sisodia, Mini Jose, Sathish Kumar, Anita Mahadevan, Narendrakumar Ramanan, Suhita Nadkarni, Deepak Nair

**Affiliations:** 1 Centre for Neuroscience, Indian Institute of Science, Bangalore 560012, India; 2 Indian Institute of Science Education and Research, Pune 411008, India; 3 Center for Molecular Neurobiology, Department of Neurobiology, The University of Chicago, IL 60637, USA; 4 Department of Neurology, University of Bonn, Bonn 53127, Germany; 5 Department of Neuropathology, NIMHANS, Bangalore 560029, India

**Keywords:** Cellular Neuroscience, Molecular Neuroscience, Optical Imaging

## Abstract

Despite intuitive insights into differential proteolysis of amyloid precursor protein (APP), the stochasticity behind local product formation through amyloidogenic pathway at individual synapses remain unclear. Here, we show that the major components of amyloidogenic machinery namely, APP and secretases are discretely organized into nanodomains of high local concentration compared to their immediate environment in functional zones of the synapse. Additionally, with the aid of multiple models of Alzheimer's disease (AD), we confirm that this discrete nanoscale chemical map of amyloidogenic machinery is altered at excitatory synapses. Furthermore, we provide realistic models of amyloidogenic processing in unitary vesicles originating from the endocytic zone of excitatory synapses. Thus, we show how an alteration in the stochasticity of synaptic nanoscale organization contributes to the dynamic range of C-terminal fragments β (CTFβ) production, defining the heterogeneity of amyloidogenic processing at individual synapses, leading to long-term synaptic deficits as seen in AD.

## Highlights

• Components of amyloidogenic machinery are organized into nanodomains
• Assembly of nanodomains differs between functional zones of the synapse
• Stochasticity of nanoscale organization dictates dynamic range of APP proteolysis
• Variability in composition of amyloidogenic machinery is associated with AD

## Graphical Abstract

**Figure:**
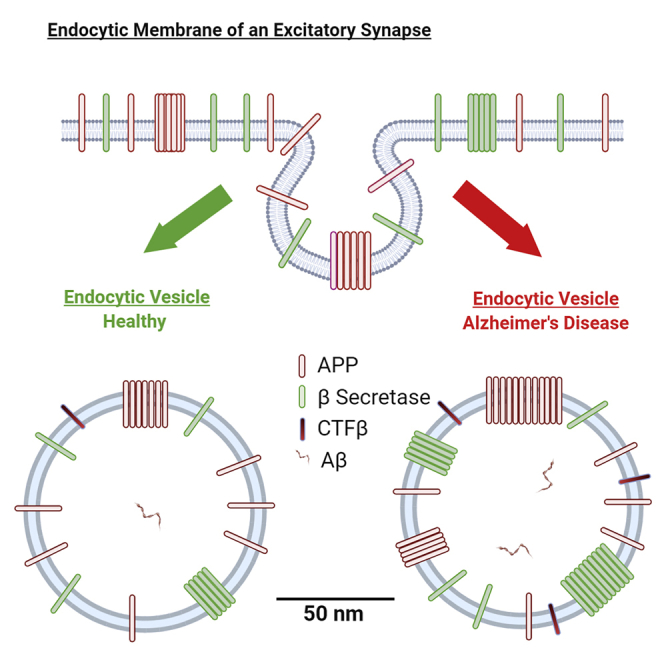

## Introduction

Enzymatic hydrolysis of peptide bonds (proteolysis) induces an irreversible alteration of the molecular structure and biological function of a protein. Proteolysis is a post-translational modification, generating functionally relevant and stable cleaved proteins referred to as proteoforms, which are pivotal regulators of many physiological and pathological processes (Klein et al., 2018; Rawlings et al., 2012). Sequential proteolysis is a targeted event where multiple enzymes act one after another on a single substrate resulting in several proteoforms (Klein et al., 2018). These alterations of the substrate molecule are controlled both spatially and temporally such that a change in the combination of proteases can result in proteoforms with antagonistic properties. Secretases are a class of proteases involved in precise proteolytic processing of amyloid precursor protein (APP), a single-pass transmembrane protein that is ubiquitously expressed throughout the body (Chow et al., 2010; Muller et al., 2017). APP can be processed by both canonical and non-canonical secretases, resulting in numerous proteoforms, mediating distinct and even opposing functions (Muller et al., 2017).

Decades of research indicate that alteration in proteolytic processing of APP is a crucial element toward progression of Alzheimer's disease (AD) (Brunholz et al., 2012; DeBoer et al., 2014; Haass et al., 2012; Sun and Roy, 2018). The major focus on APP proteolysis processing is due to its importance in the generation of a peptide proteoform referred to as amyloid beta (Aβ), an essential component of Amyloid plaques found in the brain of patients diagnosed with AD. Extensive biochemical and molecular biology studies have identified that Aβ is generated by amyloidogenic processing through sequential cleavage by β- and γ-secretases. Despite several years of focus on AD, a lack of understanding still exists on how the equilibrium is shifted toward the amyloidogenic pathway or how this shift alters the molecular machinery involved in synaptic transmission and plasticity (Montagna et al., 2017). Over the last decade, groundwork has been laid to understand AD as a disease beginning with alteration of molecular properties of individual synapses (Lesne et al., 2006; Neuman et al., 2015; Opazo et al., 2018; Wei et al., 2010; Wilhelm et al., 2014). Recent studies have indicated the subcellular segregation of APP into regulatory nanodomains on the plasma membrane in both neuronal and non-neuronal cells (de Coninck et al., 2018; Kedia et al., 2020). Further, the nanoscale fingerprints of these domains have been shown to be altered in neuronal processes and within functional subcompartments of an excitatory synapse and between different variants of APP implicated in AD (Kedia et al., 2020).

Although AD is considered to begin as a synaptopathy, it is not yet understood how the segregation of APP and secretases at nanoscale contributes toward the progression of AD (De Strooper and Karran, 2016; Haass et al., 2012; Selkoe et al., 2012; Selkoe, 2002). This is largely due to the lack of information on (1) the heterogeneity of localization of the amyloidogenic machinery within/outside functional zones of individual synapses (Harris and Stevens, 1989; Harris and Weinberg, 2012) and (2) a lack of intuitive understanding of the mechanisms that control the heterogeneity of diffusional collisions between APP and secretases resulting in different proteoforms (Ben Halima et al., 2016; Escamilla-Ayala et al., 2020; Kedia et al., 2020). Here, we have employed super-resolution imaging and analysis to reveal the subsynaptic organization of these molecules. We confirm that in addition to APP, both β- and γ-secretases are also organized into segregated domains of few tens of nanometers. This discrete association of nanodomains resemble high molecular weight multi-protein complexes with varying compositionality of secretases and APP (Chen et al., 2015; Liu et al., 2019a). We used this nanoscale heterogeneity in the molecular distribution in empirical *insilico* experiments of reconstructed vesicles to simulate the interactions between APP and secretases as diffusional collisions resulting in the product formation. We focused our efforts to understand the association of APP with β-secretases in specialized subsynaptic regions and how this stoichiometry of association directly influences the processing of APP through the amyloidogenic pathway. We present a unique data-driven realistic model for synaptic amyloidogenic processing from several thousands of synapses, wherein we identify a set of molecular determinants that decide the fate of APP proteolysis. We further demonstrate that even minor alterations in the molecular fingerprints of this synaptic nanoorganization can yield significant changes in the local product formation through amyloidogenic processing. Furthermore, with the aid of transgenic mouse models for AD and postmortem human brain tissues from AD patients, we validate the competency of this molecular model. Thus, we entail a nanoscale synaptic reaction-diffusion model of amyloidogenic processing with realistic numbers and geometrical constraints to understand molecular mechanisms that alter synaptic amyloidogenic processing.

## Results

### Differential nanoorganization of amyloidogenic proteolytic machinery in the functional domains of an excitatory synapse

The amyloidogenic processing of APP is the result of sequential proteolysis by β- and γ-secretases (Cole and Vassar, 2007; Yang et al., 2017). The spatial association of β- and γ-secretases is vital for amyloidogenic processing in different neuronal subcompartments. To comprehend this spatial variability of β- and γ-secretases within the neuronal processes and synaptic compartments, we evaluated the relative nanoscale distribution of the secretases in neuronal processes and within different functional zones of an excitatory synapse. We relied on the nanoscopic association of secretases with a marker for postsynaptic density (PSD) and a perisynaptic marker for endocytic zone (EZ), PSD95 and dynamin, respectively. Similar experimental paradigms have been previously used to segregate the fractional contribution of synaptic molecules localized to both synaptic compartments and within functional compartments of individual synapses (Kedia et al., 2020).

**Figure 1:**
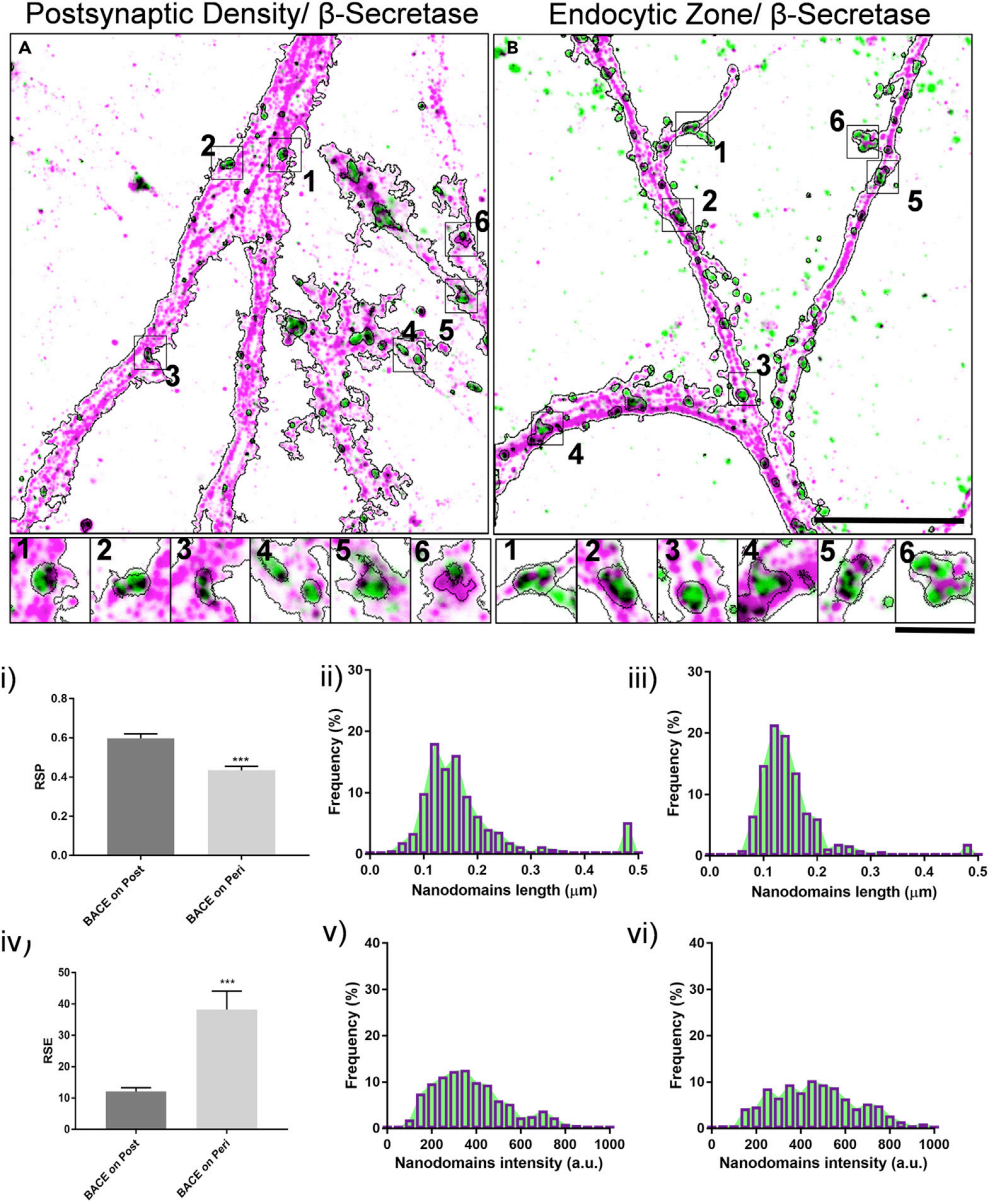
Nanoscale distribution of β-secretase in the functional zones of excitatory postsynapse using STED microscopy (A and B) Overlay of STED images of postsynaptic density marker PSD95 and a marker for endocytic zone Dynamin (green) with β-secretase (Magenta). The black contour indicates the automated detection of neuronal processes. Inset 1–6 indicate a gallery of synapses where the black contour within inset represents automatically detected regions for confocal marker for postsynapse and endocytic zone (black). Black in the overlay images represents the overlap between the corresponding green and magenta images. The scale bars in B represent 7 μm and inset corresponds to 1.4 μm. (i and iv) Comparison of RSP and RSE for quantifying colocalization of β-secretase for functional zones of an excitatory postsynapse. Data are represented as mean ± SEM. Significance was determined by unpaired two-tailed Student's t test. ∗p ≤ 0.05, ∗∗p ≤ 0.01, and ∗∗∗p ≤ 0.001, ns p > 0.05. (ii and iii) Indicate the distribution of length of all β-secretase nanodomains obtained by STED microscopy in post and perisynaptic compartments, respectively. (v and vi) Indicate the distribution of intensity of all β-secretase nanodomains obtained by STED microscopy in post and perisynaptic compartments, respectively. n = 5669 puncta (post) and 3798 puncta (peri).

The distribution of β-secretase, BACE1 was evaluated for the quantitative estimation of the association of β-secretase in PSD and EZ (Figures 1 and S1). Confocal and stimulated emission depletion (STED) microscopy were performed sequentially to evaluate the diffraction limited and nanoscale distribution of these molecules. A gallery of representative images of individual synapses obtained by confocal and STED microscopy using PSD/EZ markers and the associated molecular domains of β-secretase (nanodomain_β_) are presented (Figures 1, S1A, S1B, S1C, S1D, S1E, and S1F). The morphological and biophysical properties of nanodomain_β_ were characterized (Table 1). Interestingly, resolution scaled Pearson's (RSP) coefficient and resolution scaled error (RSE) of β-secretase in PSD and EZ were significantly different. The colocalization with β-secretase was significantly higher in PSD, while the variability was more in EZ (Figures 1i and 1iv). The nanodomain_β_ associated with PSD and EZ is referred to as nanodomain_β/PSD_ and nanodomain_β/EZ_ respectively. The distribution of length, area, intensity, and normalized intensity with respect to the median of the nanodomain_β_ intensity is indicated in Figures 1ii, 1iii, 1v, 1vi, S2i, and S2ii and Table 1.

**Table 1:** Summary of quantitative estimation of morphological and biophysical properties of different nanodomains obtained through STED microscopy

Category/parameter	Length (μm)	Area (μm^2^)	Intensity (a.u.)	Mean normalized intensity^‡^	Median normalized intensity^¶^
Nanodomain_β_	0.155 ± 0.001 (0.139,0.116–0.170)	0.0148 ± 0.0001 (0.0128, 0.0091–0.0179)	414.88 ± 3.37 (396.90,288.63–529.46)	1.00 ± 0.008	(1.00, 0.73–1.33)
Nanodomain_γ_	0.143 ± 0.001 (0.126, 0.097–0.162)	0.0129 ± 0.0001 (0.0106, 0.0063–0.0164)	283.77 ± 3.21 (228.69,106.81–407.36)	1.00 ± 0.011	(1.00, 0.47–1.78)
Nanodomain_β/PSD_	0.174 ± 0.004 (0.152, 0.121–0.194)	0.0174 ± 0.0005 (0.0148, 0.0098–0.0218)	471.90 ± 9.36 (460.70,320.80–597.70)	1.13 ± 0.022	(1.16, 0.81–1.51)
Nanodomain_β/EZ_	0.151 ± 0.003 (0.136, 0.113–0.166)	0.0141 ± 0.0004 (0.0122, 0.0087–0.0174)	381.60 ± 8.47 (355.80, 255.20–477.70)	0.92 ± 0.020	(0.90, 0.64–1.20)
Nanodomain_γ/PSD_	0.132 ± 0.002 0.112, 0.086–0.157)	0.0111 ± 0.0002 (0.0080, 0.0049–0.0147)	140.30 ± 3.12 (108.80, 77.20–162.60)	0.49 ± 0.011	(0.48, 0.34–0.71)
Nanodomain_γ/EZ_	0.158 ± 0.002 (0.142, 0.115–0.175)	0.0154 ± 0.0002 (0.0133, 0.0090–0.0187)	401.00 ± 5.52 (365.40, 254.20–513.50)	1.41 ± 0.019	(1.59,1.11–2.25)
Nanodomain_β/APP_	0.161 ± 0.003 (0.145, 0.118–0.177)	0.0158 ± 0.0003 (0.0140, 0.0096–0.0194)	398.00 ± 7.60 (371.40, 253.80–514.00)	0.95 ± 0.018	(0.94, 0.64–1.30)
Nanodomain_γ/APP_	0.129 ± 0.001 (0.116, 0.092–0.147)	0.0107 ± 0.0002 (0.0085, 0.0056–0.0134)	138.10 ± 1.85 (125.20, 88.53–170.20)	0.48 ± 0.006	(0.55, 0.38–0.74)
Nanodomain_β/γ_	0.164 ± 0.004 (0.147, 0.123–0.188)	0.0168 ± 0.0005 (0.0146, 0.0103–0.0213)	521.83 ± 11.24 (517.48, 376.88–652.30)	1.25 ± 0.027	(1.30, 0.95–1.65)

The values indicated are Mean ± SEM while values in brackets represent the median, IQR from 25% percentile to 75% percentile, ‡/¶ normalized with respect to the mean/median of the global β-secretase nanodomain intensity for nanodomain_β,_ nanodomain_β/PSD,_ nanodomain_β/EZ,_ nanodomain_β/APP,_ nanodomain_β/γ_ or with the mean/median of the global γ-secretase nanodomain intensity for nanodomain_γ,_ nanodomain_γ/PSD,_ nanodomain_γ/EZ_ and nanodomain_γ/APP_.

**Figure 2:**
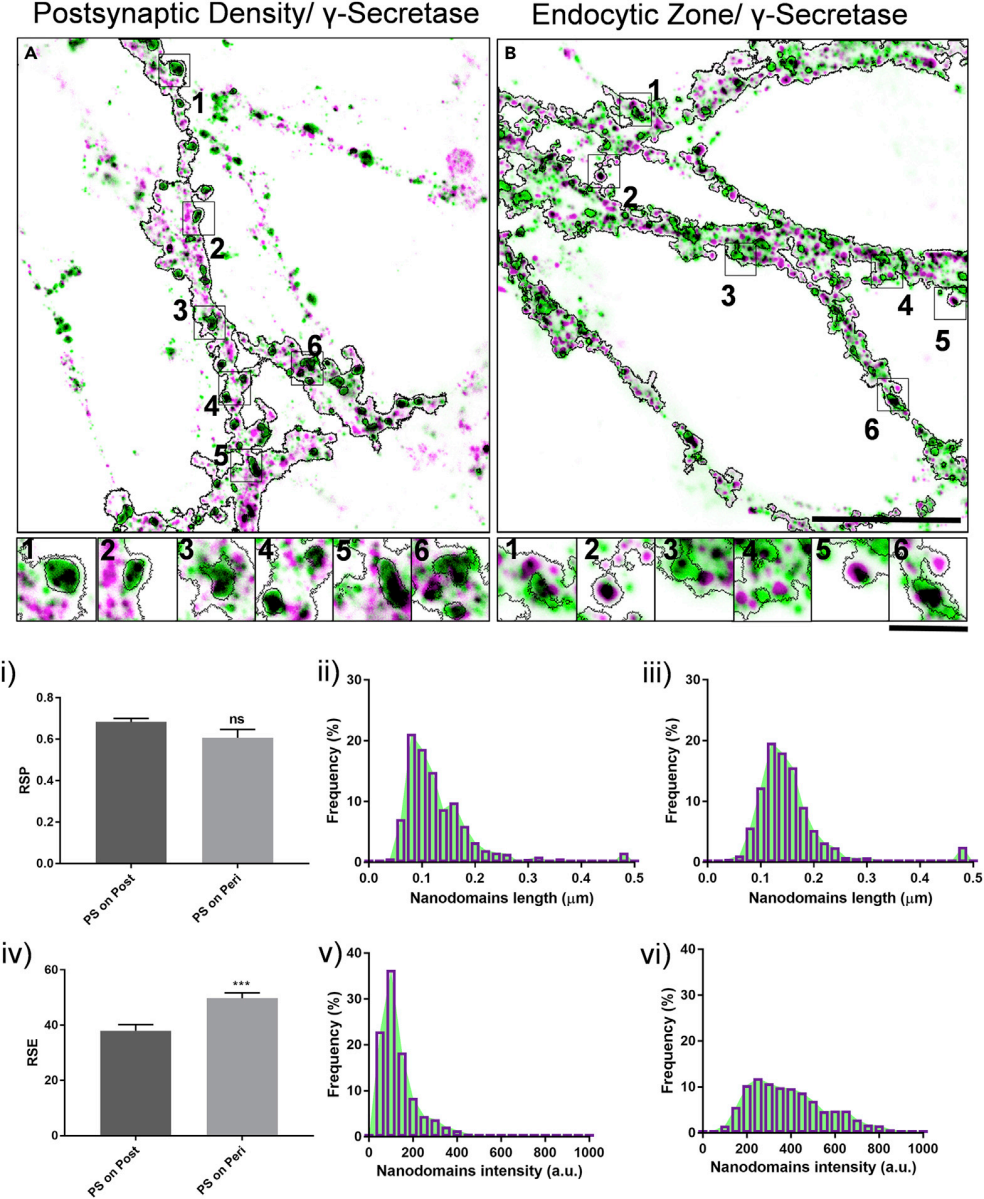
Nanoscale distribution of γ-secretase in the functional zones of excitatory postsynapse using STED microscopy (A and B) Overlay of STED images of postsynaptic density marker PSD95 and a marker for endocytic zone Dynamin (green) with γ-secretase (Magenta). The black contour indicates the automated detection of neuronal processes. Inset 1–6 indicate a gallery of synapses where the black contour within inset represents automatically detected regions for confocal marker for postsynapse and endocytic zone (black). Black in the overlay images represents the overlap between the corresponding green and magenta images. The scale bars in B represent 7 μm and inset corresponds to 1.4 μm. (i and iv) Comparison of RSP and RSE for quantifying colocalization of γ-secretase for functional zones of an excitatory postsynapse. Data are represented as mean ± SEM. Significance was determined by unpaired two-tailed Student's t test. ∗p ≤ 0.05, ∗∗p ≤ 0.01, and ∗∗∗p ≤ 0.001, ns p > 0.05. (ii and iii) Indicate the distribution of length of all γ-secretase nanodomains obtained by STED microscopy in post and perisynaptic compartments, respectively. (v and vi) Indicate the distribution of intensity of all γ-secretase nanodomains obtained by STED microscopy in post and perisynaptic compartments, respectively. n = 4936 puncta (post) and 5921 puncta (peri).

For analyzing the association of γ-secretase in PSD and EZ, the distribution of the catalytic subunit of γ-secretase namely, presenilin1 (PS1) was evaluated against PSD95 and Dynamin, respectively (Figures 2 and S3). We analyzed the PSD95 and Dynamin positive regions (Figures 2, S3A, S3B, S3C, S3D, S3E, and S3F), as well as characterized the morphological and biophysical properties of molecular domains of γ-secretase (nanodomain_γ_) in these regions (Table 1). The colocalization of γ-secretase in PSD and EZ was similar, while variability was significantly higher in EZ (Figures 2i and 2iv). The nanodomain_γ_ associated with PSD and EZ is referred to as nanodomain_γ/PSD_ and nanodomain_γ/EZ_, respectively. The distribution of length, area, intensity, and normalized intensity with respect to the median of the nanodomain_γ_ intensity is indicated in Figures 2ii, 2iii, 2v, 2vi, S4i, and S4ii and Table 1.

Our results show significant differences in the morphological and biophysical characteristics of the β- and γ-secretase nanodomains associated with PSD and EZ (Figures S2 and S4). The length, area, and intensity were significantly higher for nanodomain_β/PSD_ in comparison to nanodomain_β/EZ_. However, these parameters were significantly lower for nanodomain_γ/PSD_ when compared to nanodomain_γ/EZ._ The distribution of normalized intensity of nanodomain_β/PSD,_ nanodomain_β/EZ_ and nanodomain_γ/EZ_ showed a higher variability when compared to nanodomain_γ/PSD_ (Figures S2i and S4i). To determine the proximity of spatial association of β- and γ-secretases to PSD and EZ, we quantified nearest neighborhood distance (NND) for the secretases with respect to these functional zones. The β-secretases were proximal to PSD in comparison to EZ, while the γ-secretases associated closely with EZ (Figures S2iii and S4iii). This indicated a potential difference in the distribution of secretases associated with these domains, signifying the relevance of nanoscale association of APP, β-secretase and γ-secretase in synaptic subcompartments for the amyloidogenic processing of APP.

**Figure 3:**
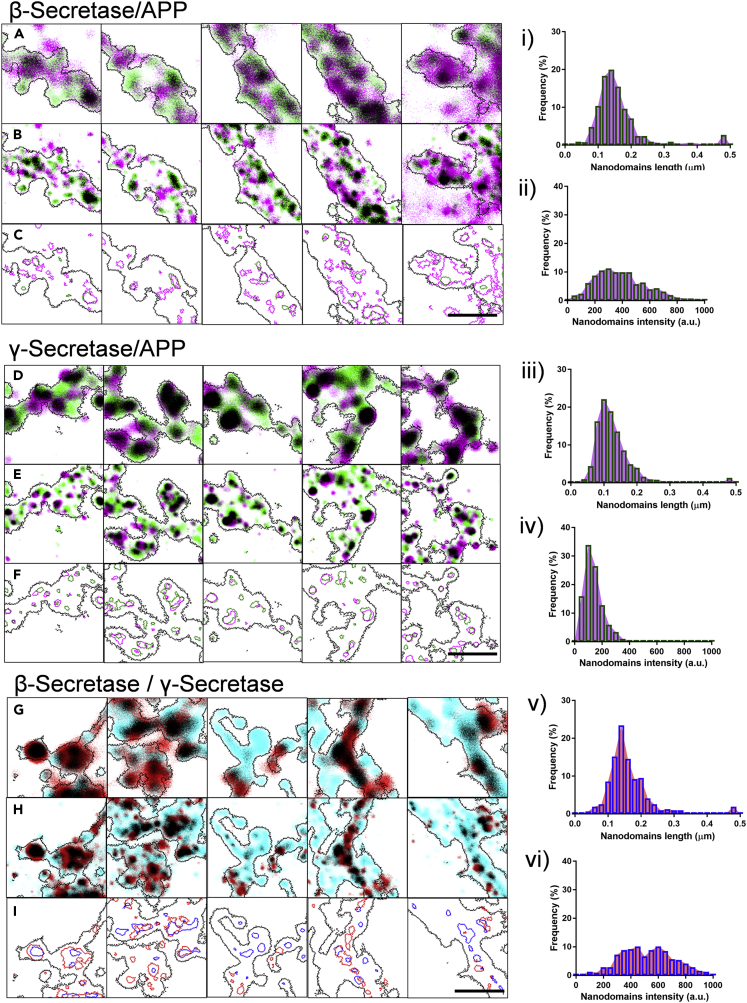
Nanoscale association of β/γ-secretase and with APP in the neuronal processes using STED microscopy (A and D) Gallery of confocal images of neuronal processes identified by automatic detection of confocal marker for APP (magenta) puncta with pseudocolour overlay of β/γ-secretase (green). (B and E) STED image of the respective regions identified from (A, D). (C and F) Represent automatically detected regions for confocal marker for β/γ-secretase (black) along with nanoscale representation of β/γ-secretase (green) and APP (magenta). Black in the overlay images represents the overlap between the corresponding green and magenta images. (i and iii) Indicate the distribution of length of all β/γ-secretase nanodomains on APP obtained by STED microscopy. (ii and iv) Indicate the distribution of intensity of all β/γ-secretase nanodomains on APP obtained by STED microscopy. (G) Confocal image of the individual γ-secretase identified by automatic detection of confocal marker for γ-secretase (red) with pseudocolour overlay of β-secretase (blue). (H) STED image of the representative regions from (G). (I) Indicates the automatically detected regions for confocal marker for β-secretase (black), nanoscale representation of β-secretase (blue) and γ-secretase (red). (v and vi) Indicate the distribution of length and intensity of all β-secretase nanodomains on γ-secretase obtained by STED microscopy, respectively. n = 13,484 puncta (β-secretase on APP), 6033 puncta (γ-secretase on APP), and 4762 puncta (for β/γ-secretases). Scale bar at (C, F, I) indicates 1.1 μm.

Further, we assessed the association of β- and γ-secretases with APP. We examined regions where APP colocalized with either β- or γ-secretase. A gallery of confocal and STED images representing this association are presented, where APP nanodomains colocalizing with β- or γ-secretases are referred to as nanodomain_β/APP_ and nanodomain_γ/APP_, respectively (Figures 3A, 3B, 3C, 3D, 3E, and 3F). The distribution of length, area, intensity, and normalized intensity with respect to the median of the nanodomain_β_ (nanodomain_β/APP_) or nanodomain_γ_ (nanodomain_γ/APP_) are presented (Figures 3i, 3ii, 3iii, 3iv, S5i, and S5ii and Table 1). Consistent with our hypothesis, the morphological and biophysical characteristics of APP associated β- and γ-secretase nanodomains were found to be significantly different (Figure S5ii). A discrete association of APP was observed with nanodomain_β_ in contrast to nanodomain_γ_, with high variability in the intensity of nanodomain_β/APP_ (Figures 3ii and 3iv) compared to nanodomain_γ/APP_ (Figures 3ii and 3iv), indicating that the concentration of β-secretase associated with the nanodomains is critical for the amyloidogenic processing of APP.

To validate this, we evaluated the relative distribution of β- and γ-secretases in synapses. We analyzed regions marked positive for β- and γ-secretases by confocal and STED microscopy (Figures 3G, 3H, and 3I). The sub-diffraction limited clusters, where β- and γ-secretases were associated are referred to as nanodomain_β/γ_. The distribution of length, area, intensity, and normalized intensity with respect to the median of the nanodomain_β_ are presented (Figures 3v, 3vi, and S5i and Table 1). Similar to nanodomain_β/APP,_ nanodomain_β/γ_ displayed a large variability in the intensity, consistent with our hypothesis that the number of β-secretase molecules associated with synaptic nanodomains might be a limiting factor for the amyloidogenic processing of APP. This was also validated by the observation that although the colocalization of the two secretases with APP remained similar, variability was higher for γ-secretases/APP compared to β-secretases/APP (Figure S5iii).

Similar to the association of secretases with functional zones of the synapse, we performed NND analysis between APP and secretases. The distance of β-secretase to APP was proximal and less variable in comparison to γ-secretases to APP and β-to γ-secretase (Figure S5iv). This further confirmed that multiple molecular parameters could define the spatial association of secretases with APP, thus directly influencing the local proteolysis of APP. APP is known to be processed sequentially by β-secretase followed by γ-secretase. The catalytic activity of β-secretase is deemed to occur in the acidic pH range (~4.5) which corresponds to membrane-bound organelles involved in the secretory pathway, such as vesicles budding from the endocytic zone of the synapse (Cole and Vassar, 2007; Lu et al., 2007; Watanabe et al., 2014). Here, quantitative parameters obtained from super-resolution microscopy on the distribution and association of APP and β/γ-secretase were used to simulate the dynamics of APP processing in the internalized membrane of endocytic zones of dendritic spines reconstructed from EM slices of CA3-CA1 region of the hippocampus.

### Nanoscale alteration of molecular fingerprints of amyloidogenic machinery in multiple models of AD

**Figure 4:**
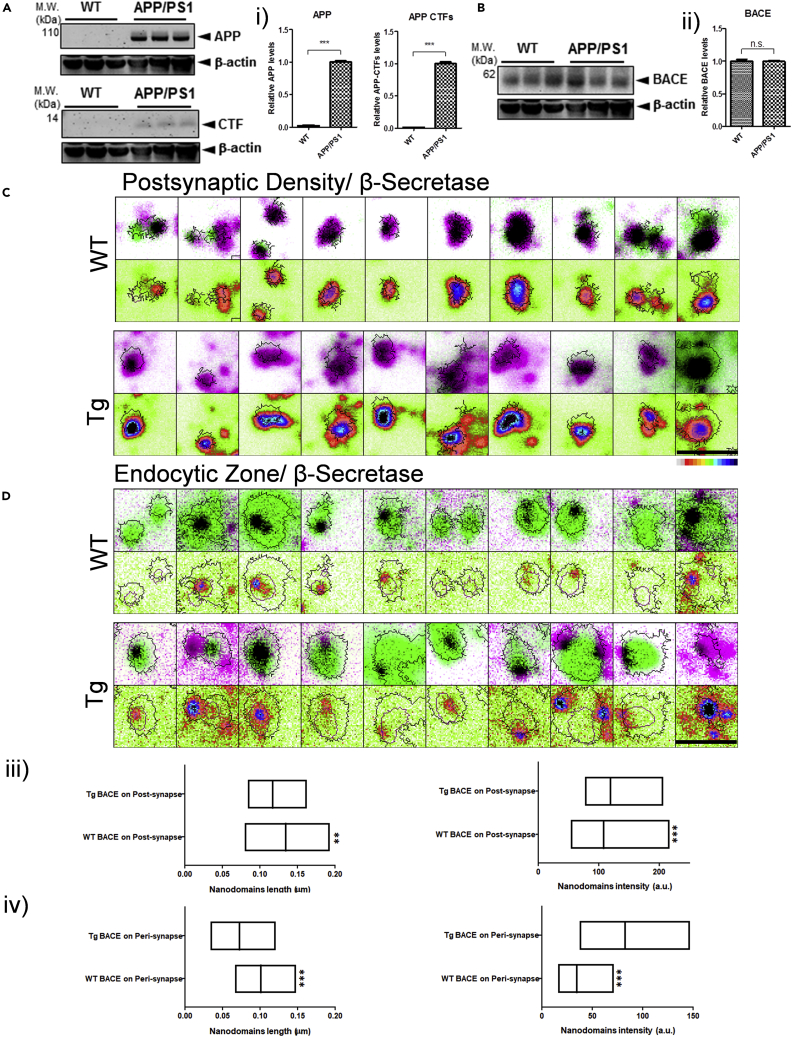
Modulation of molecular determinants for β-amyloidogenic processing in transgenic mice slices using STED microscopy (A, i, B, and ii) Representative immunoblots and densitometric quantification of APP, APP-CTFs, and BACE from WT and APP/PS1 mouse brain homogenates (n = 3). Data are represented as mean ± SEM. Significance was determined by paired two-tailed Student's t test. ∗p ≤ 0.05, ∗∗p ≤ 0.01, and ∗∗∗p ≤ 0.001, ns p > 0.0. Quantification of APP, APP-CTFs, BACE and β-actin were performed in the same immunoblot and probed with respective primary and secondary antibodies. (C and D) Nanoscale distribution of β-secretase in the post/perisynapse using STED microscopy in wild-type (WT) and APP/PS1 transgenic (Tg) mice. Post/Peri is represented as green (C/D) and BACE in magenta. The intensity of BACE is pseudocolour coded from white (minimum) to black (maximum) with black contours representing post/perisynaptic zones and purple represents PSD/EZ. Black in the overlay images represents the overlap between the corresponding green and magenta images. Scale bar at (C and D) indicate 0.75 μm. (iii and iv) Diversity in β-secretase (median/IQR 25%–75% interval) clusters for nanodomain length and intensity in post and perisynapse for WT and APP/PS1 Tg mice. n = 1585 (WT BACE on post), 1948 (Tg BACE on post), 1276 (WT BACE on peri), 1381 (Tg BACE on peri) puncta from 3 animals. Significance was determined by unpaired two-tailed Mann-Whitney test. ∗p ≤ 0.05, ∗∗p ≤ 0.01, and ∗∗∗p ≤ 0.001, ns p > 0.05.

To test the potential association of the nanoscale fingerprints of amyloidogenic machinery and AD, we evaluated the morphological and biophysical traits of β-secretase and APP in multiple models of AD. We recently reported that the lateral diffusion and nanoscale aggregation properties of APP-wildtype differed considerably from its detrimental variant namely, APP-Swedish (Kedia et al., 2020). APP/PS1 mice contain humanized APP within the Aβ region bearing the Swedish mutation, as well as PSEN1 encoding deltaE9 mutation, under the control of the mouse prion promoter (Jankowsky et al., 2001). These mice do not harbor any mutations in BACE. APP/PS1 transgenic mouse model has been well characterized for cognitive impairment (Lalonde et al., 2005; Volianskis et al., 2010), with the reduction of transient long-term potentiation by 3 months of age (Volianskis et al., 2010). Also, age-dependent loss of classical synaptic proteins such as Synaptophysin, Synaptotagmin, Homer, and PSD95 is observed in these mice as early as 4 months (Hong et al., 2016). Thus, APP/PS1 mice harboring APP-Swe mutation has been very well characterized for early synaptic deficits. In our paradigm, we selected APP/PS1 mice which were 3-4 months old. This timeline coincides with the earliest appearance of synaptic impairment which precedes the detectable levels of Aβ deposits. Thus, APP/PS1 model was chosen to evaluate the role of nanoscale synaptic deficits in the early onset of AD. Global levels of APP and C-terminal fragment (CTF) was increased in APP/PS1 (Tg) mice in comparison to wildtype (WT) due to overexpression, while the β-secretase levels remained unaltered when evaluated by immunoblotting (Figures 4A, 4B, 4i, and 4ii). Since the foresaid experiment only identifies human variant of APP, we verified the expression of the total APP pool using an antibody that recognized both human and murine APP variants (Figure S6 and Table S3) Next, we evaluated the nanoscale organization of β-secretase in different functional zones of the synapse. The quantitative association of β-secretase with PSD and EZ was assessed by imaging with STED microscopy using Shank2 and Clathrin as markers, respectively (Figures 4C and 4D). We found that the length and intensity of nanodomain_β/PSD_ and nanodomain_β/EZ_ were significantly altered between WT and Tg mice (Figures 4iii and 4iv and Table S1). Furthermore, on comparison of the integrated intensity of β-secretase in PSD/EZ (Figure S7i), we found that in Tg mice, the cumulative β-secretase levels were decreased in PSD, while it increased in EZ. Additionally, we found that the proximity of β-secretase to PSD and EZ were altered antagonistically. The NND of β-secretase to PSD increased, while a significant decrease was observed for EZ in Tg mice (Figure S7ii). Taken together, the β-secretase levels in Tg mice were augmented significantly in PSD/EZ both inside and outside of nanodomains, along with a decrease in the length of nanodomains. This nanoscale alteration of β-secretase was pronounced at EZ together with a reduction in NND, reflecting an increase in the β-secretase load per endocytic event originating from EZ in Tg mice.

**Figure 5:**
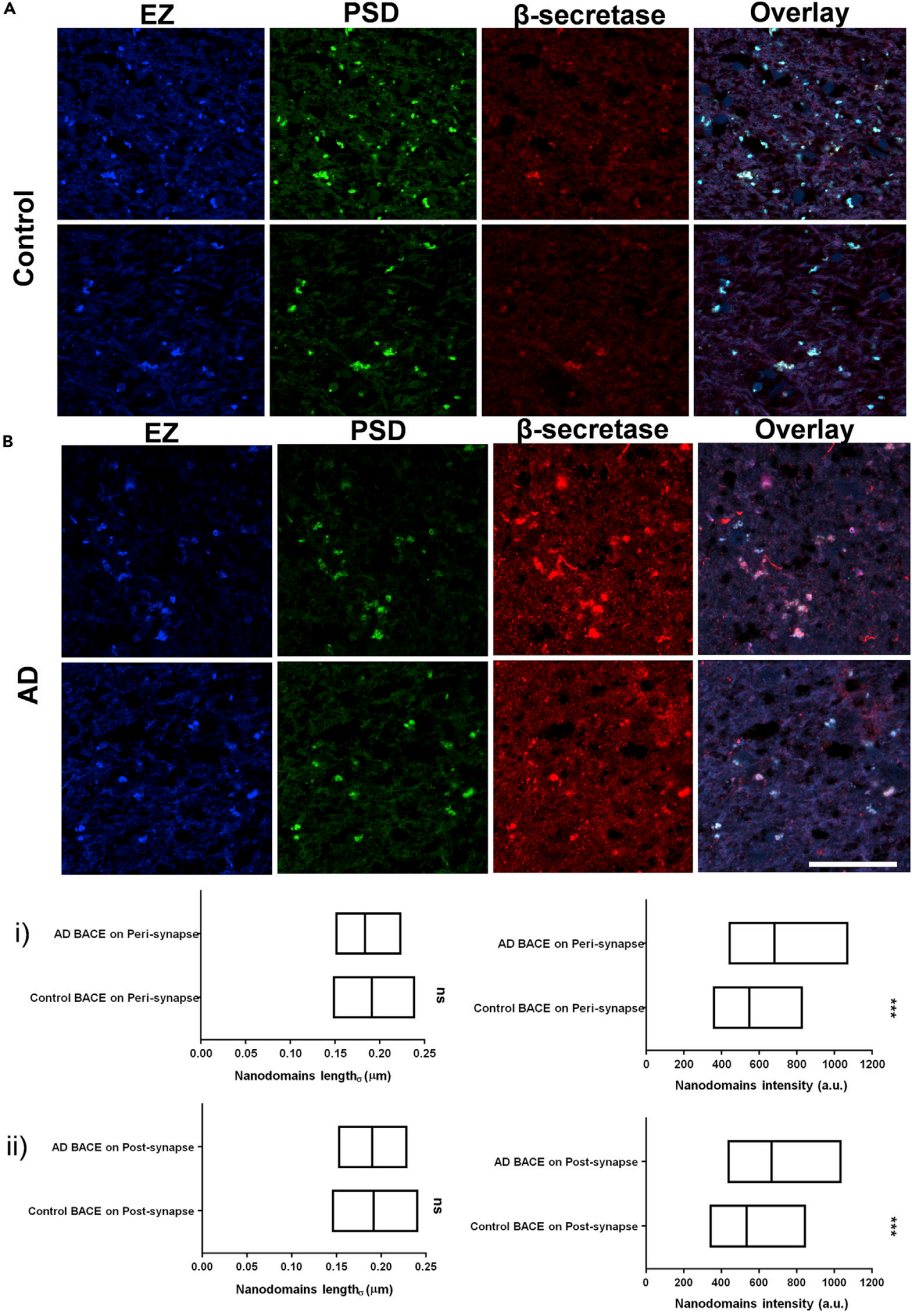
Alteration of distribution of β-secretase in human brain slices using Airyscan microscopy (A and B) Compartmentalization of β-secretase clusters in post/perisynapse in human brain slices from control (A) and AD (B) using Airyscan microscopy. White in the overlay images represents the overlap between the corresponding blue, green and red images. Scale bar at (B) indicates 30 μm. (i and ii) Diversity (median/IQR 25%–75% interval) in nanodomain length (σ) and intensity for β-secretase clusters in perisynapse (i) or postsynapse (ii) in human brain slices from AD and control represented as (median/IQR 25%–75% interval). n = 6101 (control BACE on peri), 6729 (AD BACE on peri), 5130 (control BACE on post), 4931 (AD BACE on post) puncta from 3 sets of human brains of patients with AD and their corresponding controls. Significance was determined by unpaired two-tailed Mann-Whitney test. ∗p ≤ 0.05, ∗∗p ≤ 0.01, and ∗∗∗p ≤ 0.001, ns p > 0.05. The regions depicted in (A) is the same as the regions in (Figure S8) marked as B∗ and B∗∗.

**Figure 6:**
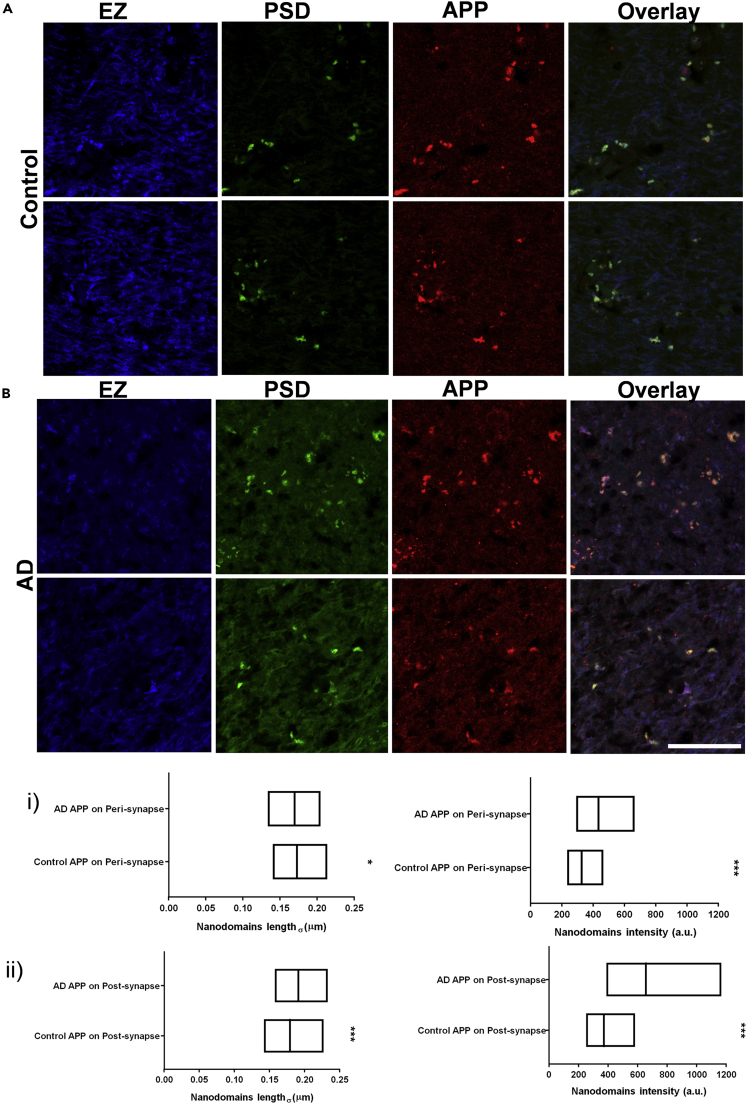
Alteration of distribution of APP in human brain slices using Airyscan microscopy (A and B) Compartmentalization of APP clusters in post/perisynapse in human brain slices from control (A) and AD (B) using Airyscan microscopy. White in the overlay images represents the overlap between the corresponding blue, green and red images. Scale bar at (B) indicates 30 μm. (i and ii) Diversity (median/IQR 25%–75% interval) in nanodomain length (σ) and intensity for APP clusters in perisynapse (i) or postsynapse (ii) in human brain slices from AD and control represented as (median/IQR 25%–75% interval). n = 7633 (control APP on peri), 8158 (AD APP on peri), 4982 (control APP on post), 5186 (AD APP on post) puncta from 3 sets of human brains of patients with AD and their corresponding controls. Significance was determined by unpaired two-tailed Mann–Whitney test. ∗p ≤ 0.05, ∗∗p ≤ 0.01, and ∗∗∗p ≤ 0.001, ns p > 0.05.

In order to evaluate if such alterations were also consistent in the human brain, we determined the length and intensity of nanodomain_APP/PSD_, nanodomain_APP/EZ_, nanodomain_β/PSD_ and nanodomain_β/EZ_ with similar markers for PSD and EZ in three sets of post-mortem human brain tissues from patients with AD and their corresponding controls (control) using Airyscan microscopy, providing sub-diffraction limited resolution (Figures S8A, S8B, S8C, and S8D, and Table S4). Similar to rodent models, we randomly chose 5–10 non-overlapping regions from the radiatum layer of the hippocampus (Figures S8E and S8F). The quantitative association of β-secretase and APP with PSD and EZ was assessed by Airyscan microscopy using Shank2 and Clathrin as markers, respectively (Figures 5 and 6). Airyscan images of β-secretase association with EZ and PSD in control and AD human brains were evaluated for the sub-diffraction limited zones of enrichment (Figures 5A and 5B). Although, there was no significant alteration in the size of nanodomain_β/PSD_ and nanodomain_β/EZ_, the content of β-secretase enriched in these domains were significantly higher in the brain of patients with AD compared to the control samples (Figures 5i and 5ii). A similar strategy was employed to characterize nanodomain_APP/PSD_ and nanodomain_APP/EZ_ in control and AD samples from the brain sections of the same patients investigated for the alteration of subsynaptic organization of β-secretase (Figures 6A and 6B). In this case, the APP content in the nanodomains increased significantly in both EZ and PSD (Figures 6i and 6ii). However, comparing AD samples to the control, the size of the nanodomain_APP/PSD_ increased in contrast to a small yet significant decrease in the size of the nanodomain_APP/EZ_ (Figures 6i and 6ii).

Next, to understand heterogeneity in organization of amyloidogenic machinery at finer spatial scales, we performed STED microscopy on one set of age- and gender-matched AD and control samples to investigate if we could enhance the spatial separation between EZ and PSD, and performed an extensive analysis of the nanodomains of β-secretase and APP associated with these functional zones (Figures S9A, S9B, S9C, and S9D and Table S4). We found that the intensity of nanodomains of APP and β-secretase in both PSD and EZ increased in AD (Figures S9i and S9ii and Table S1). While the length of nanodomains of APP increased in PSD, it was reduced in EZ in AD (Figures S9i and S9ii and Table S1). In contrast to APP, the length of nanodomains of β-secretase increased in EZ, while remaining similar at PSD in AD (Figures S9i and S9ii and Table S1). The investigations related to the biophysical characteristics of the nanodomain_APP/PSD_, nanodomain_APP/EZ,_ nanodomain_β/PSD_ and nanodomain_β/EZ_ between AD and control remained consistent with our observations with Airyscan microscopy, confirming the robustness of the results. On comparing the integrated intensity of APP and β-secretase in PSD/EZ observed by STED (Figures S10i and S10ii), the cumulative APP and β-secretase levels were found to be increased in EZ in AD, compared to PSD. Interestingly, APP levels were augmented in PSD while β-secretase levels decreased in PSD in AD (Figures S10i and S10ii). NND of APP and β-secretase did not differ significantly in the PSD, while at EZ it was reduced for β-secretase and augmented for APP (Figures S10iii and S10iv). These observations in humans reinforce our observations in Tg mice, confirming higher β-secretase levels at EZ leading to an increased load of β-secretase per endocytic event. Altogether, these lines of evidence reveal the alteration of nanoscale compositionality of β-amyloidogenic machinery in AD pathogenesis.

### *In silico* experiments in unitary vesicles predict the biophysical determinants of CTFβ production

Recent studies have indicated unhindered diffusion of APP on the membrane in contrast to a confined motion in functional nanodomains of APP (nanodomain_APP_), as well as differential localization of APP molecules in PSD and EZ (Kedia et al., 2020). Concurrent to the observation of secretases that we report here, an EZ can have different permutations and combinations of components of β-amyloidogenic machinery. To estimate physiologically the realistic dynamics of CTFβ production, we systematically simulated the diffusion of APP molecules on a membrane that includes, (1) single molecule mobility and (2) reduced mobility represented by nanodomain_APP_ of varying content (Table S2 (Kedia et al., 2020)). These simulations were carried out in a confined volume representative of a unit endocytic process. This reconstructed endosomal compartment where the activity of β-secretase was optimal (diameter 0.120 μm) is referred to as a “unitary vesicle”. The characteristics of unitary vesicles are shown in Table S2.

**Figure 7:**
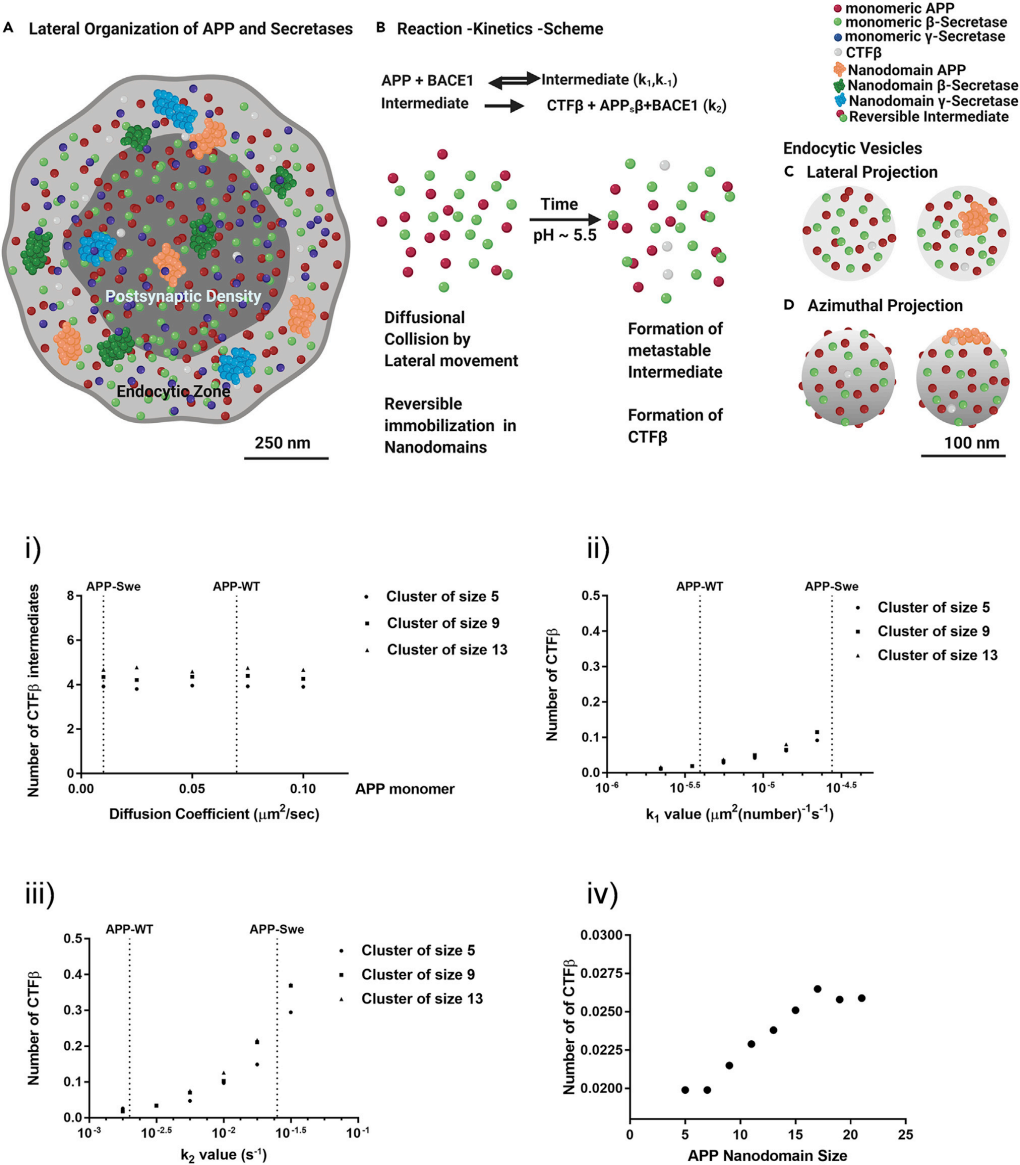
*Insilico* evaluation of CTFβ production for APP-WT/Swe show differential processing kinetics within unitary vesicles (A) Schematic of nanoscale lateral organization of components of amyloidogenic machinery indicating the variability of free and segregated molecules of APP, β- and γ-secretases in the functional Zones of an excitatory synapse. Scale bar at A and D indicates 250 and 100 nm respectively. (B) Two-step reaction model for amyloidogenic processing of APP by β-secretase through lateral diffusion and single molecule collisions. The collision can either result in a metastable intermediate or process APP into CTF fragment which will stay on the membrane. Lateral diffusion can aid in the formation or elimination of APP nanodomains on the membrane. (C and D) Representative illustration of lateral (C) and azimuthal (D) view of two vesicles originating from different regions of the endocytic zone. The vesicles are illustrated either with the presence or absence of a nanodomain of APP, where the β secretases and extra-nanodomain APP molecules are diffusively distributed. (i) The number of CTFβ intermediates formed in each endocytic vesicle as a function of experimentally observed diffusion coefficients for APP-WT/Swe. The diffusion coefficients (median) corresponding to APP-WT and APP-Swe are demarcated by a vertical dotted line. The intermediate product formation is not affected by the rate of diffusion of APP within a vesicle. All other parameters were kept constant for this plot. (ii) The number of CTFβ formed in an endocytic vesicle as a function of forward reaction rate for forming CTFβ intermediates (k_1_). (iii) Amount of CTFβ formed in an endocytic vesicle as a function of irreversible forward reaction rate for intermediates to products (k_2_). The other simulation parameters such as diffusion coefficients (median of APP-WT) and reaction rates are kept constant and set to APP-WT values, while k_1_ and k_2_ are varied independently in (ii) and (iii) respectively. CTFβ production substantially increases with increase in both k_1_ and k_2_. CTFβ formation is more sensitive to variations in k_2_ than k_1_ (for APP-WT conditions). (iv) Probability to produce CTFβ is correlated with the APP nanodomain size internalized per unitary vesicle. The results obtained for APP-Swe is indicated in Figure S11.

An immediately relevant question in this context was how the spatial distribution and biophysical characteristics of a simulated unitary vesicle affect CTFβ production. The resulting number of CTFβ in the reaction is a result of competing dynamics of reaction rates, diffusion and numbers of reactants and geometrical constraints of the unitary vesicle. We investigated two contrasting hypotheses as advantageous for the production of CTFβ. We asked if (1) slow-moving-large and densely populated APP clusters enabled more productive encounters with β-secretase (referred to as “the sitting duck hypothesis”) or (2) rapidly diffusing APP monomers (referred to as ‘movers and shakers hypothesis’). We simulated APP and β-secretase binding on a unitary vesicle for the physiologically realistic range of mobility parameters and typical numbers of each species (Table S2). The binding rates for the kinetic scheme (Figures 7A and 7B) were modified from those measured independently in experiments ((Ben Halima et al., 2016), refer to methods for calculation of binding rates). The simulations were carried out for unitary vesicles with APP clusters of sizes 5, 9, and 13 as illustrative instantiations of clusters seen in vesicles. For simplicity, BACE1 was always simulated as a monomer in these control “*in silico”* experiments. The complete distribution of cluster sizes and an entire range of diffusion coefficients from APP-WT to APP-Swe within and outside the nanodomains have been characterized and described previously (Kedia et al., 2020). These values are summarized in Table S2. The simulated single molecule diffusion rates were an order of magnitude faster than nanodomain_APP_ and was consistent with experimental observations (Figures 7 and S11 and Table S2). The simulation also included the distribution of single molecules and nanodomains as seen in Figures 7C and 7D. For the unitary vesicles, the mobility of the β-secretase was considered similar to APP monomers. The freely diffusing β-secretase monomers interacted with APP molecules to produce CTFβ (Figure 7i). Each simulation was carried out for 5 s to mimic the time scales over which endocytosis takes place. The simulation results suggested that the production of intermediates was not sensitive to the possible range of rates of diffusion (including both APP-WT and APP-Swe) inside unitary vesicles (Figures 7i and S11i).

Next, we quantified the differences in the production of CTFβ corresponding to changes in k_1_ and k_2_ in APP-WT and APP-Swe conditions (Figures 7ii, 7iii, S11ii, and S11iii). A significant biophysical property that distinguishes APP-Swe from APP-WT is the reaction rate of intermediate product formation and CTFβ production. Both the forward rates k_1_ and k_2_ were seen to be higher for APP-Swe (Ben Halima et al., 2016). The number of CTFβ formed while varying k_1_ but keeping the diffusion coefficients, APP densities and k_-1_ and k_2_ as per APP-WT is shown in Figure 7ii. The number of CTFβ formed by varying k_2_ but keeping all other parameters; diffusion coefficients, k_1_ and k_-1_ as per the APP-WT is shown in Figure 7iii. In comparison with APP-WT, forward reaction rate k_1_ for APP-Swe led to as much as 7 times increase in CTFβ production (Figure S11ii). When both k_1_ and k_2_ values for APP-Swe were instantiated, we observed that CTFβ formation was more sensitive to changes in k_2_ than in APP-WT rate conditions (Figures S11ii and S11iii).

### Probability of CTFβ production increased with confined APP/β-secretase inside nanodomain within a unitary vesicle

In the data shown in Figure S12, the total number of APP molecules on the unitary vesicle was observed to increase with the size of the nanodomain. The increased number of total APP associated with larger cluster sizes was an outcome of the high affinity of cluster formation and slow diffusion of a cluster, effectively allowing a vesicle with a cluster to capture more APP. The nanodomain_APP_ cluster size was simulated with experimental values obtained for APP confinement values within EZ (Kedia et al., 2020). Our simulations showed that most vesicles with an increased amount of APP confinement invariably ended up producing more CTFβ molecules than vesicles without an APP cluster, validating the sitting duck hypothesis (Figure 7iv). The data for the APP-Swe are shown in Figure S11iv. We also simulated unitary vesicles with a uniform distribution of freely diffusive APP molecules, while the number of β-secretase molecules immobilized inside nanodomain_β_ was linearly varied. We observed that the CTFβ production is correlated with the size of nanodomain_β_ (Figure S13i). When we performed the same simulations with APP-Swe, there was a substantial increase in the production of CTFβ molecules compared to APP-WT (Figure S13i). Our simulations support the observations of enhanced clustering of β-secretase molecules in multiple models of AD (Figures 4, 5, S9, and S13i). In the case where unitary vesicles had both nanodomain_APP_ and nanodomain_β_, elevated levels of CTFβ was maintained (data not shown). The propensity to form CTFβ in this case was comparable to a unitary vesicle with nanodomain_β_. In summary, the increase in the rate of CTFβ production was correlated with an increase in the content of APP or β-secretase in nanodomain_APP_ or nanodomain_β_. This was also consistent with our observations of augmented levels of Aβ42 in primary hippocampal neurons of APP/PS1 Tg mice compared to that of the wildtype (Figures S14A and S14B). Here, both the average and integrated content of Aβ42 was significantly enhanced in Dynamin enriched compartments (Figure S14i). In these simulations, clusters of various sizes were instantiated, and their binding affinities were selected as per the APP-WT affinity values. Each simulation was carried out for 5 s and CTFβ present at the end of each simulation was considered (see Transparent Methods and Table S2 for details). Our simulations showed up to a 20–25% increase in CTFβ formation for vesicles with an increased size of nanodomain_APP_ or nanodomain_β_. When mimicking the unitary vesicle with the forward reaction rates corresponding to APP-Swe, there was a multi-fold increase in the probability of production of CTFβ (Figures S11iv and S13i). These simulations predict that inside the confined volume of a unitary vesicle where the molecular density of APP and β-secretase are close to realistic estimation, the forward reaction rates to produce CTFβ take a dominant role in amyloidogenic processing.

## Discussion

Over the last decade, several studies have shown that individual synapses are heterogeneous structures where nanoscale segregation of synaptic molecules on the membrane plays a crucial role in synaptic transmission and plasticity (Chen et al., 2018; Dani et al., 2010; Nair et al., 2013; Venkatesan et al., 2020). Despite the functional overlap of amyloidogenic machinery, such evaluations on its subsynaptic organization have not yet been addressed in detail (Almeida et al., 2005; Muller et al., 2017; Snyder et al., 2005). Here, we show the nanoscale compartmentalization and differential association of components of β-amyloidogenic machinery in functional zones of the excitatory synapse. We have identified that within the functional zones of the synapse (Harris and Weinberg, 2012), the constituents of this machinery are clustered into nanoscale structures called nanodomains. The nanodomains of APP, β- and γ-secretases overlap discretely at neuronal processes with varying compositionality. These nanodomains and their association is similar to mega-Dalton sized secretase complexes which contain necessary molecules involved in the proteolysis of APP (Chen et al., 2015; Liu et al., 2019a). Consistent with this observation, these secretase rich complexes were also found to be very heterogeneous in their composition in neuronal synapses. The heterogeneity in composition is a cumulative outcome of (i) morphological and biophysical properties of the nanodomains, (ii) the distribution of the freely diffusible pool and nanodomains in the functional zones of synapse, and (iii) the proximity of nanodomain of APP to secretases. The molecular signatures that we identified varied between functional zones of the synapse, as well as between individual synapses. This implied that the variability in compositionality directly impacts the association/dissociation of these domains, thus controlling the locus of APP processing.

Recent evidences indicate that APP and γ-secretase molecules are transiently immobilized on the plasma membrane with the existence of an equilibrium between the nanodomain and extra-nanodomain APP and secretase molecules (Escamilla-Ayala et al., 2020; Kedia et al., 2020). The existence of APP nanodomains in neuronal and non-neuronal cells have been confirmed (de Coninck et al., 2018; Kedia et al., 2020). However, the segregation of γ-secretase into nanodomains in non-neuronal cells is still a matter of debate (Escamilla-Ayala et al., 2020; Liu et al., 2019a). The results presented here are consistent with previous reports of γ-secretase segregated into nanodomains in the synaptic compartments of neurons (Schedin-Weiss et al., 2016). Additionally, immobilization kinetics, morphology and the amount of APP molecules immobilized in nanodomains differed between well characterized wild-type (APP-WT), detrimental (APP-Swedish) and protective (APP-Icelandic) variants of APP (Kedia et al., 2020). All these parameters influence the availability of APP and secretases molecules per unit area on the neuronal membrane. The spatial proximity of β-amyloidogenic machinery is a pre-requisite for the proteolysis of APP. Since the spatial availability of components of amyloidogenic machinery is a limiting factor in the sequential cleavage of APP, we evaluated the heterogeneity in the localization by investigating the compositionality within subsynaptic compartments. We found a high variability in the intensity of β-secretase nanodomain with PSD, EZ, APP and with γ-secretase. The γ-secretase nanodomains were tightly associated with APP and PSD and were variable at EZ. This implied that in addition to the availability of β-secretase, how these secretase molecules associate with different pools of APP in the functional compartments of the synapse is a limiting factor for amyloidogenic processing of APP. Our results on the nanoscale variability of β-secretase are consistent with various evidences which associate a minor but enzymatically active portion of β-secretase with γ-secretase in functional complexes of high molecular weight resulting in the generation of Aβ (Liu et al., 2019a). However the mechanism of this association is unclear since only around a maximum of 10% of the total cellular β-secretase seems to be incorporated into such high molecular weight complexes resulting in a rather high variability of colocalization between β- and γ-secretases (Liu et al., 2019a, 2019b).

APP, β- and γ-secretases are transmembrane molecules which are distributed heterogeneously both in the functional compartments of synapses and on the neuronal membrane. Although the segregation of these molecules happens in both PSD and EZ, their environment and local distribution determine the association between them. This heterogeneity can be regulated by (1) molecular organization of APP and secretases within EZ at the time of internalization and (2) competing timescales for APP processing and diffusional collisions of these molecules on the membrane. As described previously, the synaptic endosomes of the postsynaptic compartment are derived from the membrane of the EZ (Watanabe et al., 2014), where APP and secretases can coexist either in nanodomains or outside as a diffusive pool. This implies that a vesicle recycled from EZ can have either a diffusive population or a mix of diffusive population recruited along with regulatory nanodomains of APP and secretases. Thus, the amount of APP processed through an endocytic compartment is dictated by the compositionality of the individual compartments. These pools of APP/secretases or nanodomains of APP or secretases in varying combinations result in a dynamic range of Aβ production at the level of individual synapses. To include these dynamics, we instantiated the compositionality of amyloidogenic machinery in endocytic compartments, where only the compositionality of APP was altered. We systematically investigated if the “Sitting duck hypothesis” wherein APP molecules are confined in nanodomain_APP_ would be favorable for APP processing over the “Movers and shakers hypothesis” wherein single APP molecules are diffusing randomly. Interestingly, within the confined volume of the unitary vesicle, physiologically relevant changes in diffusion of molecules did not have a significant effect on the formation of CTFβ. The current estimation of secretases in the vesicle puts them at a very high concentration on the vesicular membrane. At such a high concentration in a confined volume, the diffusive effects of APP can be a minor factor in the production of CTFβ. Previous observations on the proteolysis of APP in multisecretase complexes have confirmed the formation of Aβ. Although the mechanism remains unclear, it poses an alternative to overcome product formation through stochastic collision of molecules on the plasma membrane. Thus, the spatial association of these complexes might regulate the local processing of APP resulting in an instantaneous increase in the rate of production of Aβ, as observed by increased molecular presence of components of amyloidogenic machinery in nanodomains during the progression of AD.

The processing of APP by secretases in the unitary vesicle is considered as a two-step reaction model, where a reversible intermediate complex is formed that can further result in the formation of CTFβ with an irreversible forward rate of reaction. Previous observations have indicated differences in rate of reaction of both APP-WT and APP-Swe (Ben Halima et al., 2016). It was, therefore, important to address the implication of these differences on APP processing by secretases. Within a unitary vesicle, the irreversible forward rate of reaction showed a significant effect on CTFβ production in comparison to the rate of formation of reversible intermediate product upon collision. Our data showed that within a unitary vesicle, the total number of APP molecules internalized per process was positively correlated with the probability of occurrence of nanodomain_APP_. Subsequently, as the amount of APP and/or β-secretase immobilized in nanodomains increased, there was a significant increase in the amount of CTFβ being produced. The high affinity of collisions and cluster formation of APP and β-secretase molecules within a unitary vesicle ensured this arrangement. Through super-resolution imaging and analysis, it was observed that the secretase molecules were also found in nanodomains inside EZ. It remains elusive how acidic intraluminal pH within unitary vesicles can lead to altered kinetics of diffusional collisions between APP and β-secretase molecules. The propensity to form clusters of APP and β-secretase is found to be increased in multiple models of AD; our simulation demonstrates how it can contribute to the molecular progression of AD. A potential alternate mechanism could be that APP, β- and γ-secretase clusters could dissociate into monomers when exposed to acidic pH in the lumen, increasing the probability to collide with its substrate molecules. However, this remains to be experimentally verified and thus remains an open question.

Here, we have characterized molecular determinants that control the rate of formation of products in ascending order: diffusion of molecules, size of the nanodomain, the onward reaction rate of formation of reversible APP-secretase complex and the forward reaction rate for formation of CTFβ from the APP-secretase complex. This was consistent with the diffusive behavior of APP-Swe (Kedia et al., 2020) and with the biochemical rate of reaction of the formation of CTFβ (Ben Halima et al., 2016). Though diffusion itself does not have a notable influence within the unitary vesicle, it may have an important role on the synaptic membrane where the lateral diffusion of transmembrane molecules can control their local density through nanoscale association and segregation in short time scales. Several scaffolding molecules are known to interact at various levels with components of the β-amyloidogenic machinery (Perreau et al., 2010; Sisodia and St George-Hyslop, 2002). It would be interesting to see if the confinement kinetics of APP or secretases are affected by these molecules. Since the number of APP and secretases molecules in the synaptic compartment is very high, the retrieval of these molecules by endocytosis would happen in a timescale of seconds to minutes (Kumari et al., 2010; Lu et al., 2007; Watanabe et al., 2014). These variables confirm that individual synapses have their own dynamic range for Aβ production arising from the molecular composition of the EZ membrane. This is in line with our observation of higher Aβ42 content in Dynamin enriched compartments, and is also consistent with the previous reports of increased Aβ content in both synaptic and endosomal compartments during the molecular progression of AD (Abramov et al., 2009; Gouras et al., 2010; Pickett et al., 2016; Sannerud et al., 2011, 2016).

Most of the modulators have been shown to affect APP processing and alter the profile of the Aβ peptides (De Strooper et al., 2010; Liu et al., 2019b). In a recent study, a close evaluation of multiple BACE inhibitors indicated that they extended the protein's half-life (Liu et al., 2019b). This would generate additional Aβ or process neuronal substrates different from APP, affecting both synaptic function and non-amyloidogenic processing in long-term. The molecules that could dissociate β/γ secretase complexes (e.g. roburic acid) without altering the secretase levels would be better suited to diminish the amyloidogenic processing of APP (Liu et al., 2019b). Thus, we envision controlling the molecular properties of APP and secretases such as the association and dissociation of the nanoclusters or ability to control their lateral exchange instead of modulating the enzymatic properties of the secretases which could be the focus for next generation therapeutic targets (Kedia and Nair, 2020).

APP and secretases are present in multiple compartments of neurons and are known to interact among themselves as well as with several other molecules crucial for synaptic maintenance and neuronal function (Chen et al., 2015; De Strooper et al., 2010; Haapasalo and Kovacs, 2011; Muller et al., 2017). The selective interactions with these molecules could alter the localization and availability of APP and secretases involved in both amyloidogenic and non-amyloidogenic pathways. It is also known that association of APP among themselves and its family of molecules can influence the generation of Aβ (Eggert et al., 2018; Gorman et al., 2008; Khalifa et al., 2010). In addition to the foresaid variables, post-translational modifications of APP and secretase as well as its association with lipid rafts is known to interfere with the rate of formation of different proteoforms (Grimm et al., 2017; Hicks et al., 2012; Rajendran and Annaert, 2012). However, the intracellular conditions that decide the probability of occurrence of specific pathways could be determined by the availability of molecules at a specific functional zone of the synapse. It remains to be seen how each of these interactions could contribute to the local trafficking and confinement rates that would decide the nature of rate of formation of both canonical and non-canonical proteoforms of APP.

Our study sheds light on how the changes in the nanoorganization of amyloidogenic machinery at PSD and EZ could contribute to the alterations in dynamic load of amyloidogenic processing. The local increase in concentration of APP and secretases directly influences the load of these molecules in each vesicle, contributing to the overall increase in Aβ production. Moreover, a gradual increase in such an association would also be able to influence endocytic processes occurring distant from synapses, that could affect the production of Aβ not only in synapses but also in extra-synaptic compartments. Finally, the existence of mega-Dalton rich complexes containing machinery for canonical and non-canonical processing of Aβ has already shown to generate Aβ *in vitro* (Liu et al., 2019a). Our study confirms that at least few of these nanodomains could be a potential locus for these mega-Dalton-rich complexes with the ability to influence Aβ production. Though, the mechanism of Aβ generation in these complexes remains vague, it would still support the existence of different Aβ pools.

Further, recent observations demonstrate the nanoscale architecture of human Aβ plaques revealing a dense core with a peripheral halo (Querol-Vilaseca et al., 2019). The spatial elevation of APP within nanodomains would complement either the amyloidogenic or non-amyloidogenic processing of APP. This local increase has the potential to create a nucleus for forming a dense core with higher order Aβ species aggregating in specific patterns with higher packing density. In such a scenario, the molecular density would be higher at the center and the unorganized binding and/or unspecific aggregation of the smaller Aβ structures outside would be loosely bound, creating the halo for the observed dense core of plaques. The recent technical advances to evaluate the nanoscale aggregation properties of soluble and insoluble proteins *in vivo* and *in vitro* would allow us to examine this in real-time at molecular scales (Balzarotti et al., 2017; Sezgin et al., 2019). Combination of high-resolution studies with labeling strategies enabling better detection of subcellular structures in 3D and super-resolution imaging of endogenous molecules tagged by single cell gene editing strategies would provide insights on the nanoscale variability of chemical reactions at single excitatory synapses (Kedia et al., 2020; Nishiyama et al., 2017; Tang et al., 2016; Willems et al., 2020). These observations with minimal perturbations on the signaling pathways would enable us to better understand the mechanistic intricacies of the molecular pathology underlying the neurodegenerative diseases such as AD.

The different models used to evaluate the nanoscale organization of β-amyloidogenic machinery converge to provide evidence that the compositionality of this machinery is altered at synapses, and is a critical determinant in deciding the shift in equilibrium toward β-amyloidogenic pathway. This is in resonance with our investigations in multiple models of AD, wherein we show an increase in the content of β-secretase and APP in nanodomains. This support our hypothesis that the availability of β-secretase and APP in nanodomains of subsynaptic compartments can be a limiting or a contributing factor for β-amyloidogenic processing of APP. Each synapse integrates to form a system that is regulated by both local and global homeostasis, where the local signature of the machinery becomes the decisive factor for β-amyloidogenic processing. Altogether, we describe a holistic approach for the systematic investigation of AD as a synaptopathy. This approach uncovers a fundamental nanomachinery, where alteration in real-time molecular interactions in the scale of milliseconds to minutes can contribute toward long-term deficits such as those seen in AD.

### Limitations of the study

We focused our observations based on 2D super-resolution imaging paradigms. Though the resolution is improved laterally, we believe a finer paradigm that improves axial resolution can be used such as 3D super-resolution imaging. In addition to this, employing volumetric labeling in combination with 3D super-resolution imaging would define the geometrical representation of sub neuronal structures with better accuracy. Secondly, APP/PS1 mutant mice expresses humanized APP within Aβ region (that bears the Swedish mutation, APP-Swe) along with PS1ΔE9. Both are expressed under the control of the mouse prion promoter (Jankowsky et al., 2001). We believe that the use of better models where transgenes are not overexpressed or selective enrichment of neurons with single gene editing methods would allow us to quantify the molecular changes happening precisely at individual synapses. The final limitation of the study is associated with modeling, where we used the mobility and immobilization kinetics of APP molecules to mimic the reaction-diffusion kinetics in the vesicles. The mobility of β-secretase molecules was assumed to be similar to APP. These parameters could vary in membrane bound compartments where the intraluminal pH is acidic in nature. It is an open question if the endocytosed clusters would remain as such or would break apart inside the vesicles. Despite these predictions, both the cases (presence of a cluster or dissociation of cluster into monomers) would result in elevated CTFβ levels in membrane bound compartments.

### Resource availability

#### Lead contact

Further information and requests for resources and reagents should be directed to and will be fulfilled by the lead contact, Deepak Nair (deepak@iisc.ac.in).

#### Materials availability

The study did not yield any new reagents.

#### Data and code availability

The study did not generate any additional data sets other than provided in the manuscript.

## Methods

All methods can be found in the accompanying Transparent Methods supplemental file.

## Supplemental information

Document S1. Transparent methods, Figures S1–S14, and Tables S1–S4
