# Supplementary material for: Alteration in synaptic nanoscale organization dictates amyloidogenic processing in Alzheimer's disease
Source: iScience. 2020 Dec 11;24(1):101924. doi: 10.1016/j.isci.2020.101924 (PMC7773964; doi:10.1016/j.isci.2020.101924)
Supplement: Document S1. Transparent methods, Figures S1–S14, and Tables S1–S4 [file mmc1.pdf]

iScience, Volume *24*

Supplemental Information

Alteration in synaptic nanoscale organization dictates amyloidogenic processing in Alzheimer's disease

Shekhar Kedia, Pratyush Ramakrishna, Pallavi Rao Netrakanti, Nivedita Singh, Sangram S. Sisodia, Mini Jose, Sathish Kumar, Anita Mahadevan, Narendrakumar Ramanan, Suhita Nadkarni, and Deepak Nair

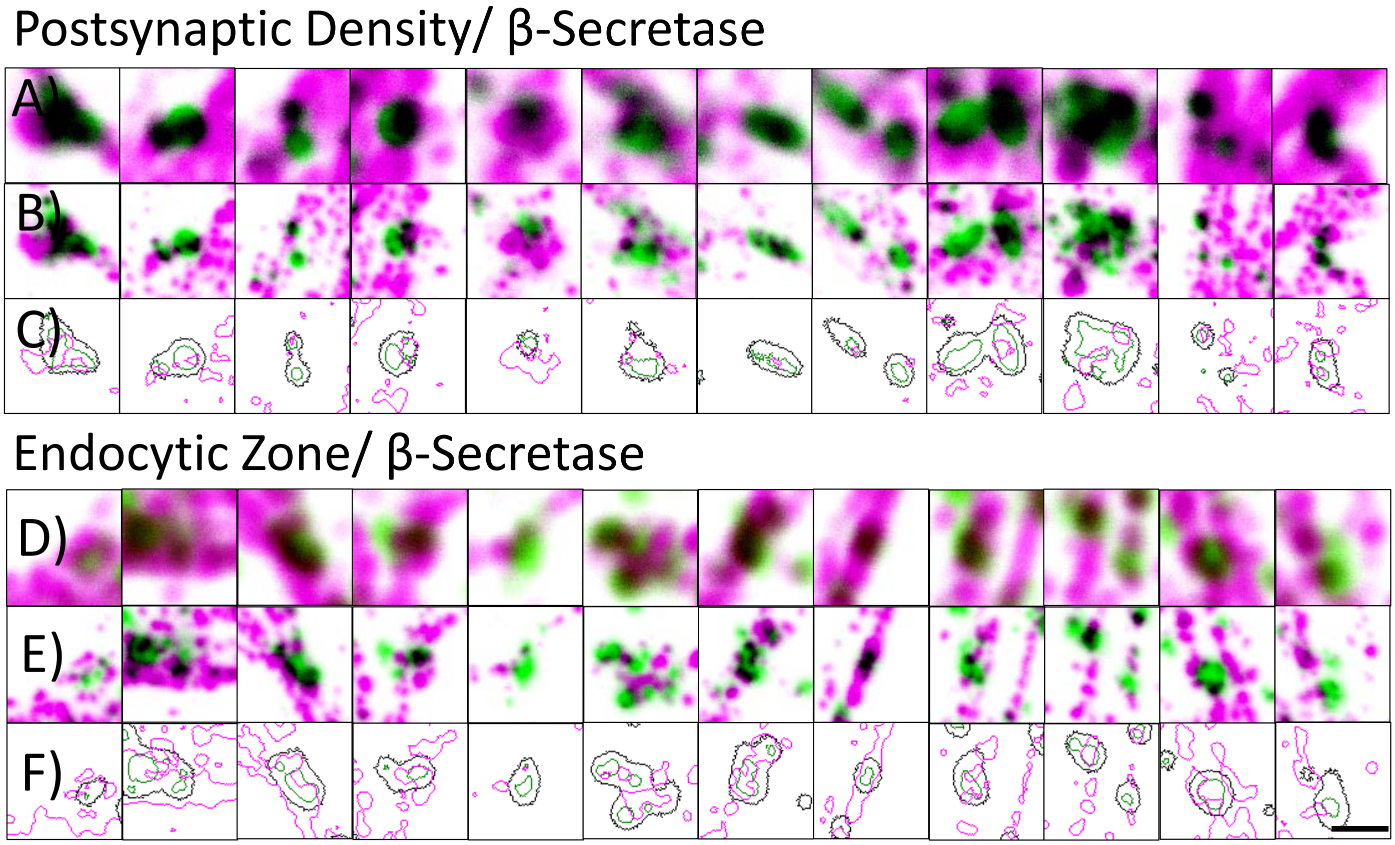

Figure S1. Nanoscale distribution of β-secretase in functional zones of an excitatory post-synapse. Related to Figure 1: (A) A gallery of confocal images of the individual synapses identified by automatic detection of postsynaptic marker PSD95 (green) with pseudocolour overlay of β-secretase (magenta). (B) STED image of the same synapses identified in (A). (C) Represent automatically detected regions for confocal marker identified for postsynaptic marker (black), PSD (green) and β-secretase (magenta). (D) A gallery of confocal images of the regions identified by automatic detection of perisynaptic marker Dynamin (green) puncta with pseudocolour overlay of β-secretase (magenta). (E) STED image of the regions identified in (D). (F) Represent automatically detected regions for confocal marker identified for perisynaptic compartment (black), EZ (green) and β-secretase (magenta). Black in the overlay images represents the overlap between the corresponding green and magenta images. Scale bar at (F) indicates 0.6 µm.

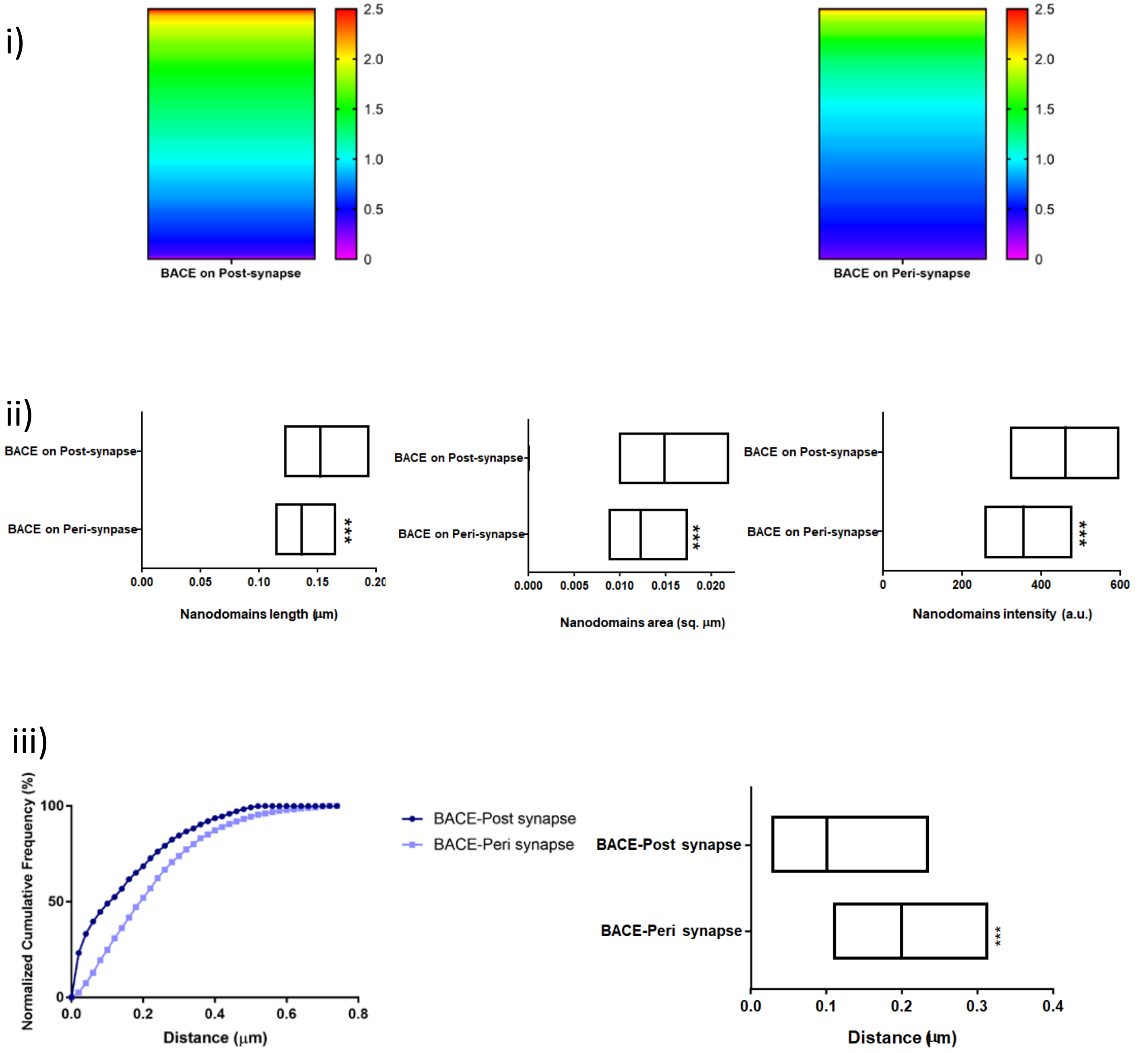

Figure S2. Nanoscale architecture of β-secretase clusters in different functional zones of a synapse using STED microscopy. Related to Figure 1 and Table 1: (i) (left to right) A heatmap of the nanodomain intensity of β-secretase in post and perisynapse normalized with respect to the median of the global β-secretase nanodomain intensity. (ii) (left to right) Diversity (median/IQR 25% to 75% interval) in β-secretase clusters for nanodomain length, area and intensity in post and perisynapse. (iii) (left to right) Indicate the distribution (left) and diversity (median/IQR 25% to 75% interval) (right) in observed nearest neighbor distances from β-secretase to post/perisynapse. Significance was determined by unpaired two-tailed Mann–Whitney test. n= 5669 puncta (post) and 3798 puncta (peri). *P ≤ 0.05, **P ≤ 0.01, and ***P ≤ 0.001, ns P> 0.05.

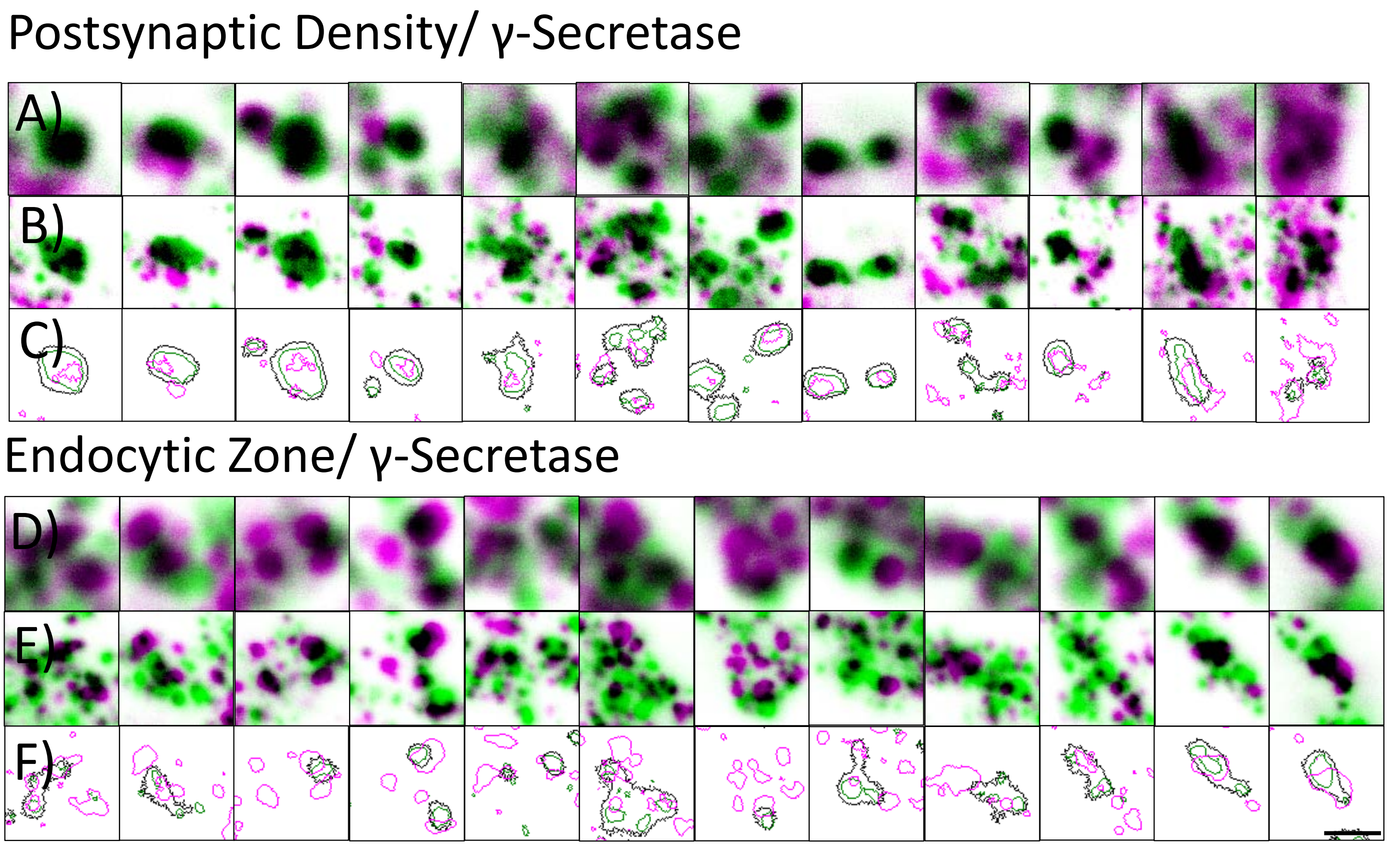

Figure S3. Nanoscale distribution of γ-secretase in functional zones of an excitatory post-synapse. Related to Figure 2: (A) A gallery of confocal images of the individual synapses identified by automatic detection of postsynaptic marker PSD95 (green) with pseudocolour overlay of γ-secretase (magenta). (B) STED image of the same synapses identified in (A). (C) Represent automatically detected regions for confocal marker identified for postsynaptic marker (black), PSD (green) and γ-secretase (magenta). (D) A gallery of confocal images of the regions identified by automatic detection of perisynaptic marker Dynamin (green) puncta with pseudocolour overlay of γ-secretase (magenta). (E) STED image of the regions identified in (D). (F) Represent automatically detected regions for confocal marker identified for perisynaptic compartment (black), EZ (green) and γ-secretase (magenta). Black in the overlay images represents the overlap between the corresponding green and magenta images. Scale bar at (F) indicates 0.6 µm.

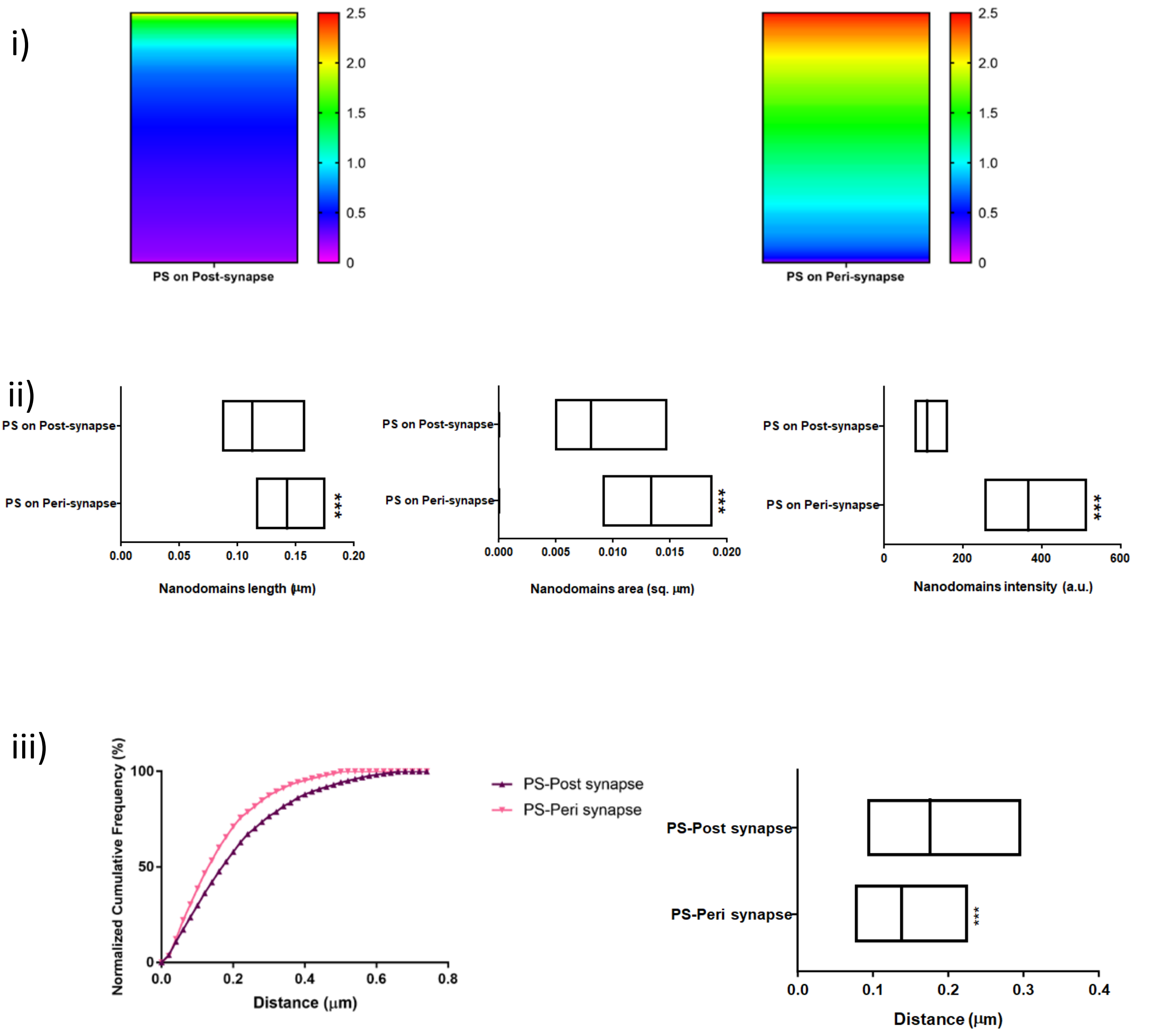

Figure S4. Nanoscale architecture of γ-secretase clusters in different functional zones of a synapse using STED microscopy. Related to Figure 2 and Table 1: (i) (left to right) A heatmap of the nanodomain intensity of γ-secretase in post and perisynapse normalized with respect to the median of the global γ-secretase nanodomain intensity. (ii) (left to right) Diversity (median/IQR 25% to 75% interval) in γ-secretase clusters for nanodomain length, area and intensity in post and perisynapse. (iii) (left to right) Indicate the distribution (left) and diversity (median/IQR 25% to 75% interval) (right) in observed nearest neighbor distances from γ-secretase to post/perisynapse. Significance was determined by unpaired two-tailed Mann–Whitney test. n= 4936 puncta (post) and 5921 puncta (peri). *$P \leq 0.05$, **$P \leq 0.01$, and ***$P \leq 0.001$, ns $P > 0.05$.

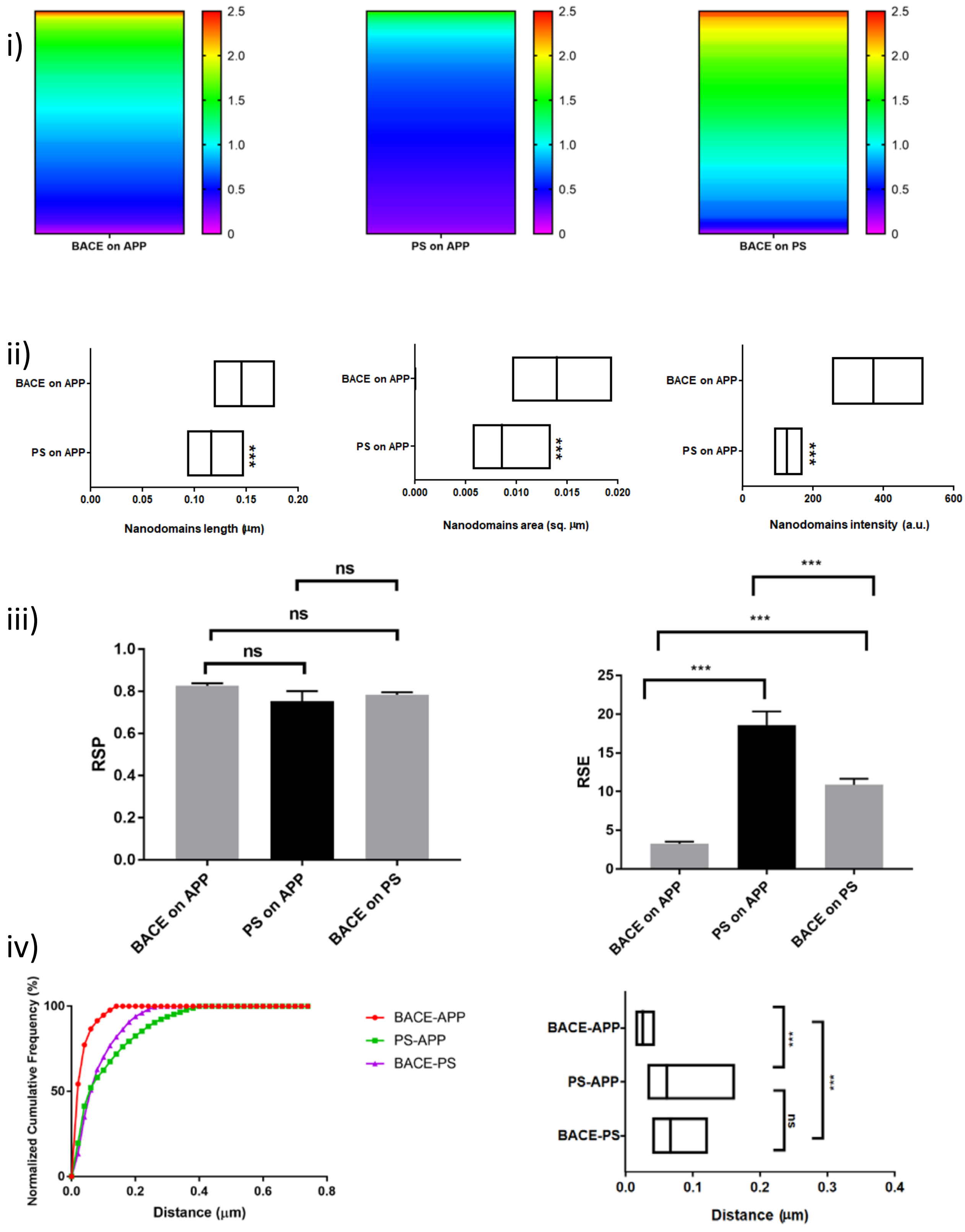

Figure S5. Nanoscale architecture of β/γ-secretase clusters with APP in the neuronal processes using STED microscopy. Related to Figure 3 and Table 1: (i) (left to right) A heatmap of the nanodomain intensity of β/γ-secretase clusters with APP normalized with respect to the median of the global β-secretase nanodomain intensity (BACE on APP and BACE on PS) or with the median of the global γ-secretase nanodomain intensity (PS on APP). (ii) (left to right) Diversity (median/IQR 25% to 75% interval) in β/γ-secretase clusters with APP in neuronal processes for nanodomain length, area and intensity. Significance was determined by unpaired two-tailed Mann–Whitney test. (iii) Comparison of RSP and RSE for quantifying colocalization of β/γ-secretase and with APP in neuronal processes. Data are represented as mean +/- SEM. Significance was determined by one-way ANOVA followed by Tukey's multiple comparison test. (iv) (left to right) Indicate the distribution (left) and diversity (median/IQR 25% to 75% interval) (right) in observed nearest neighbor distances from β-secretase to APP, γ-secretase to APP and β-secretase to γ-secretase. Significance was determined by Kruskal-Wallis test followed by Dunn's multiple comparison test. n= 13484 puncta (β-secretase on APP), 6033 puncta (γ-secretase on APP) and 4762 puncta ((β-secretase on γ-secretase). *P ≤ 0.05, **P ≤ 0.01, and ***P ≤ 0.001, ns P> 0.05.

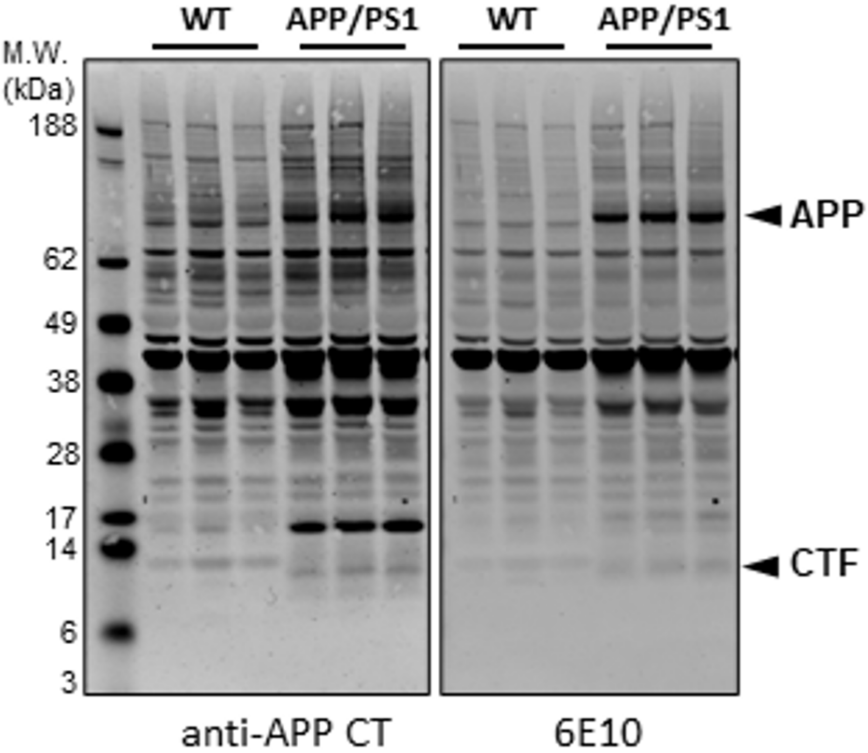

Figure S6. Global levels of APP and C-terminal fragments (CTFs) is increased in APP/PS1 (Tg) mice in comparison to wildtype (WT) mice. Related to Figure 4: SDS-PAGE and western blotting of the transgenic (APP/PS1) and non-transgenic (WT) mouse brain homogenates immunoblotted with anti-APP antibody (CT15) which recognizes both murine and human APP variants and with 6E10 antibody which recognizes only human APP variants shows the overexpression of APP in the transgenic (APP/PS1) as compared to non-transgenic (WT) mice. In addition to APP, higher levels of APP-CTFs are also observed in the transgenic mice (APP/PS1) due to APP overexpression.

Mice

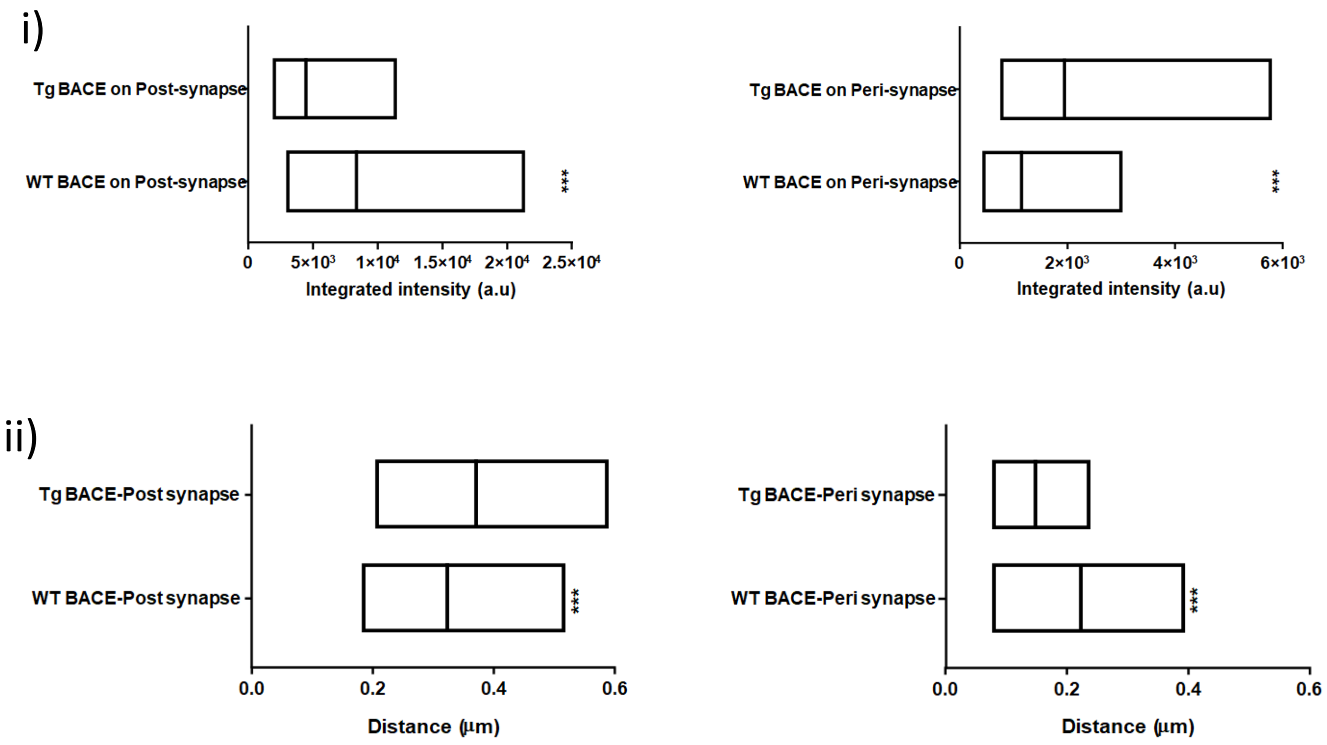

Figure S7. Compartmentalization of β-secretase in different functional zones of the synapse in mice brain slices using STED microscopy. Related to Figure 4: (i) (left to right) Diversity in β-secretase (median/IQR 25% to 75% interval) integrated intensity in post and perisynapse for WT and APP/PS1 Tg mice. (ii) (left to right) Diversity (median/IQR 25% to 75% interval) in observed nearest neighbor distances from β-secretase to post/perisynapse for WT and APP/PS1 Tg mice. n= 1585 (WT BACE on post), 1948 (Tg BACE on post), 1276 (WT BACE on peri), 1381 (Tg BACE on peri) puncta from 3 animals. Significance was determined by unpaired two-tailed Mann–Whitney test. *P ≤ 0.05, **P ≤ 0.01, and ***P ≤ 0.001, ns P > 0.05.

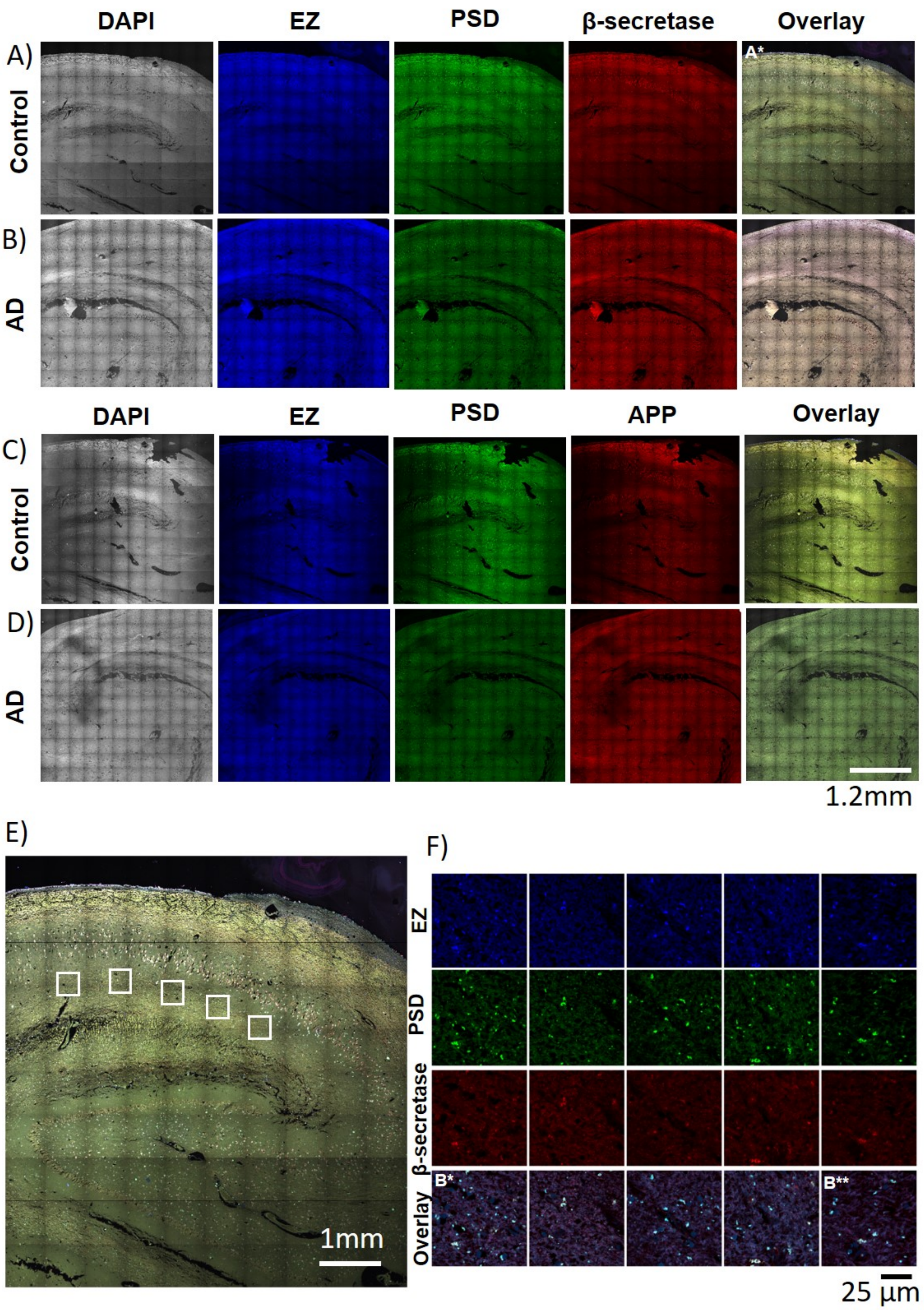

Figure S8. Heterogeneous distribution of components of β-amyloidogenic machinery in human brain slices using Airyscan microscopy. Related to Figure 5 and 6: (A, B, C, D) Visualization of a section of human brain immunostained for a marker for post/perisynapse with β-secretase (A, B) or APP (C, D) from control (A, C) and AD (B, D) using Airyscan microscopy. (F) Magnified view of the boxed regions in (E, same region marked as A*) showing discrete localization of β-secretase in different functional zones of the synapse. White in the overlay images represents the overlap between the corresponding blue, green and red images. Scale bar at (D) indicates 1.2 mm, at (E) 1 mm and at (F) 25 µm. The regions depicted in B* and B** is the same as the regions in Fig. 5A.

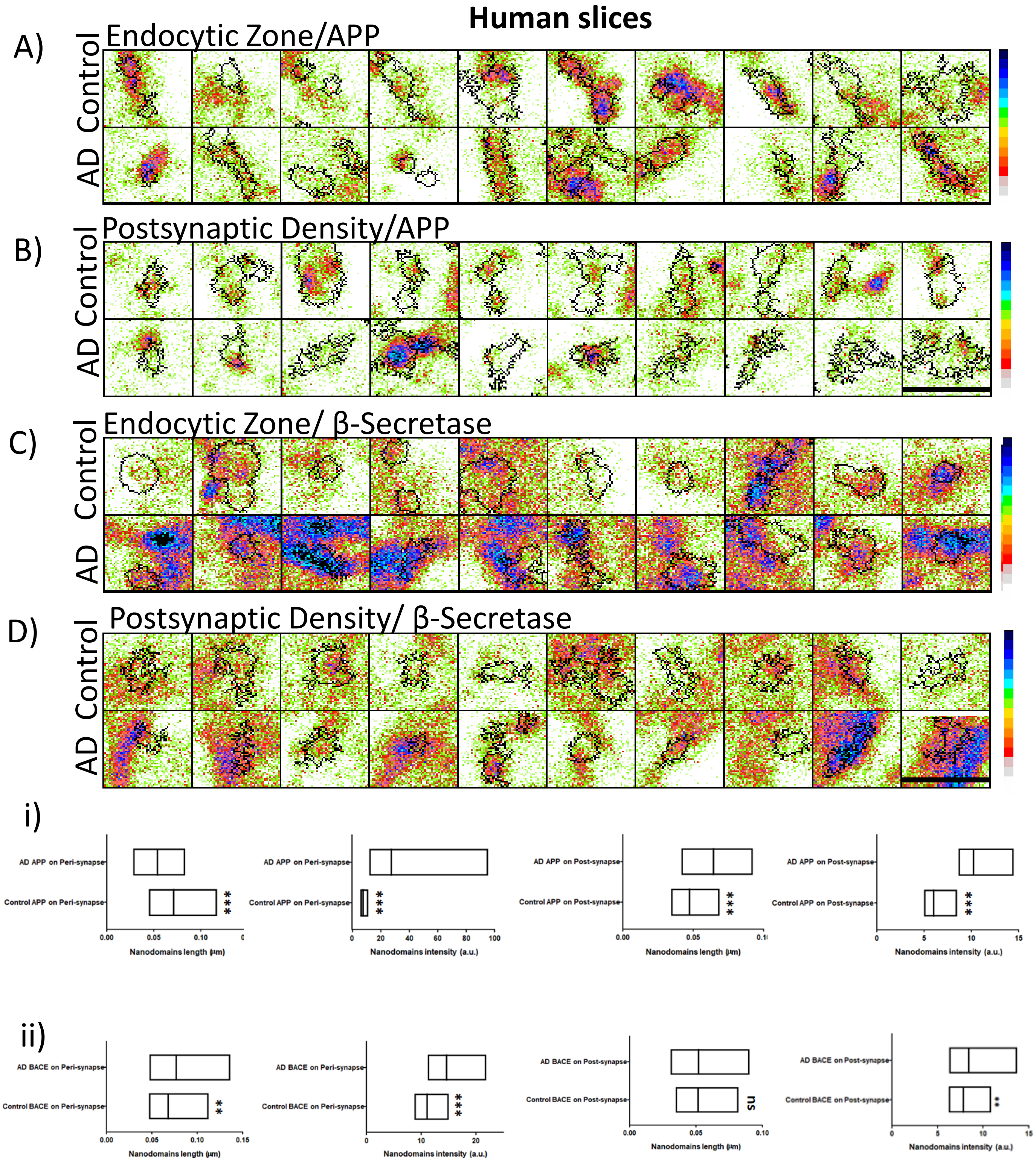

Figure S9. Alteration of nanoscale molecular determinants involved in β-amyloidogenic machinery in human brain slices using STED microscopy. Related to Figure 5 and 6: (A, B, C, D) Nanoscale colocalization of APP (A, B) and β-secretase (C, D) clusters in post/perisynapse in human brain slices from AD and control using STED microscopy. The intensity of APP and BACE is pseudocolour coded from white (minimum) to black (maximum) with black contours representing PSD/EZ. Scale bar in B and D indicates 0.75 µm. (i, ii) Diversity in nanodomain length and intensity for APP and β-secretase clusters in post/perisynapse in human brain slices from AD and control represented as (median/IQR 25% to 75% interval). n= 1637 (control APP on post), 1197 (AD APP on post), 1574 (control APP on peri), 1733 (AD APP on peri), 2461 (control BACE on post), 1539 (AD BACE on post), 1627 (control BACE on peri), 1595 (AD BACE on peri) puncta from 1 set of human brain of patient with AD and their corresponding control. Significance was determined by unpaired two-tailed Mann–Whitney test. *$P \leq 0.05$, **$P \leq 0.01$, and ***$P \leq 0.001$, ns $P > 0.05$.

Human

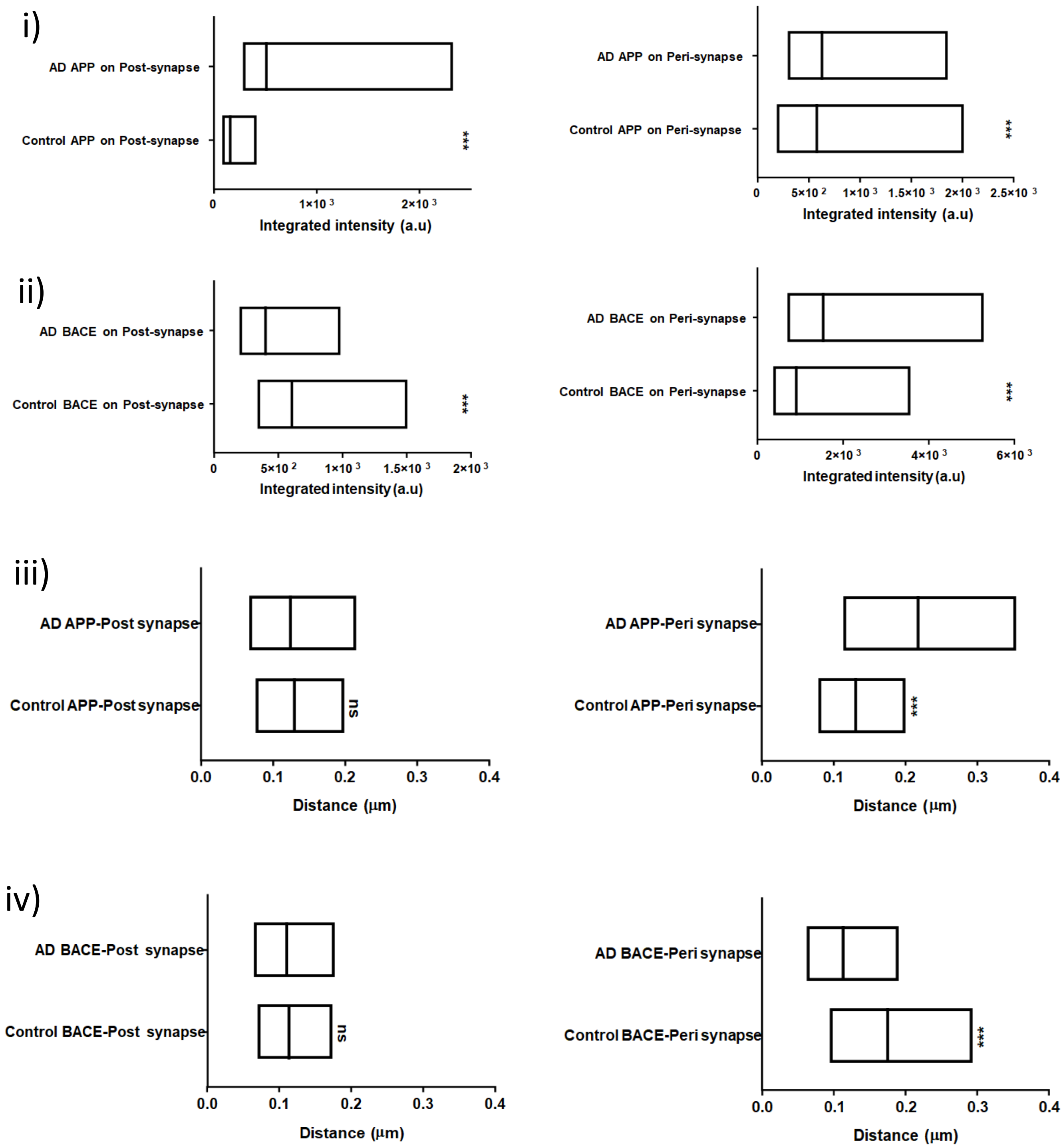

Figure S10. Compartmentalization of APP/β-secretase in different functional zones of a synapse in human brain slices using STED microscopy. Related to Figure 5 and 6: (i, ii) Diversity in APP (i) and β-secretase (ii) integrated intensity in post/perisynapse (left to right) in human brain slices from AD and control represented as (median/IQR 25% to 75% interval). (iii, iv) Diversity (median/IQR 25% to 75% interval) in observed nearest neighbor distances from APP/β-secretase to post/perisynapse (left to right) in human brain slices from AD and control. n= 1637 (control APP on post), 1197 (AD APP on post), 1574 (control APP on peri), 1733 (AD APP on peri), 2461 (control BACE on post), 1539 (AD BACE on post), 1627 (control BACE on peri), 1595 (AD BACE on peri) puncta from 1 set of human brain of patient with AD and their corresponding control. Significance was determined by unpaired two-tailed Mann–Whitney test. $^{*}P \le 0.05$, $^{**}P \le 0.01$, and $^{***}P \le 0.001$, ns $P > 0.05$.

APP Swe

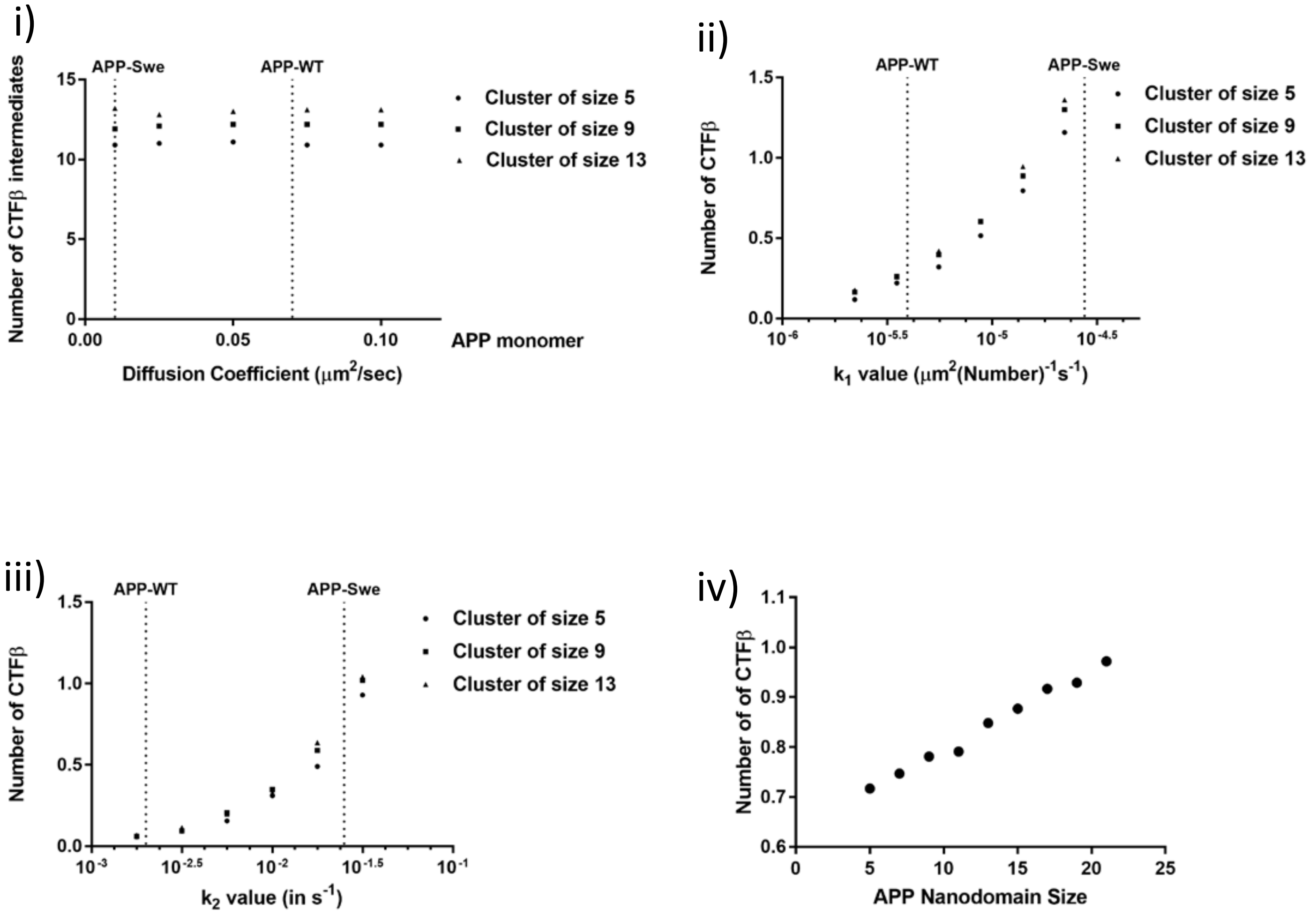

Figure S11. *Insilico* evaluation of CTFβ production for APP-Swe show differential processing kinetics within unitary vesicles. Related to Figure 7: (i) The number of CTFβ intermediates formed in each endocytic vesicle as a function of experimentally observed diffusion coefficients for APP-Swe. The diffusion coefficients (median) corresponding to APP-WT and APP-Swe are demarcated by a vertical dotted line. The intermediate product formation is not affected by the rate of diffusion of APP within a vesicle. All other parameters were kept constant for this plot. (ii) The number of CTFβ formed in an endocytic vesicle as a function of forward reaction rate for forming CTFβ intermediates (k_1). (iii) Amount of CTFβ formed in an endocytic vesicle as a function of irreversible forward reaction rate for intermediates to final products (k_2). The other simulation parameters such as diffusion coefficients (median of APP-Swe) and reaction rates are kept constant and set to APP-Swe values, while k_1 and k_2 are varied independently in (ii) and (iii) respectively. CTFβ production substantially increases with increase in both k_1 and k_2. CTFβ formation is more sensitive to variations in k_2 than k_1 (for APP-Swe conditions). (iv) Probability to produce CTFβ is correlated with the APP nanodomain size internalized per unitary vesicle.

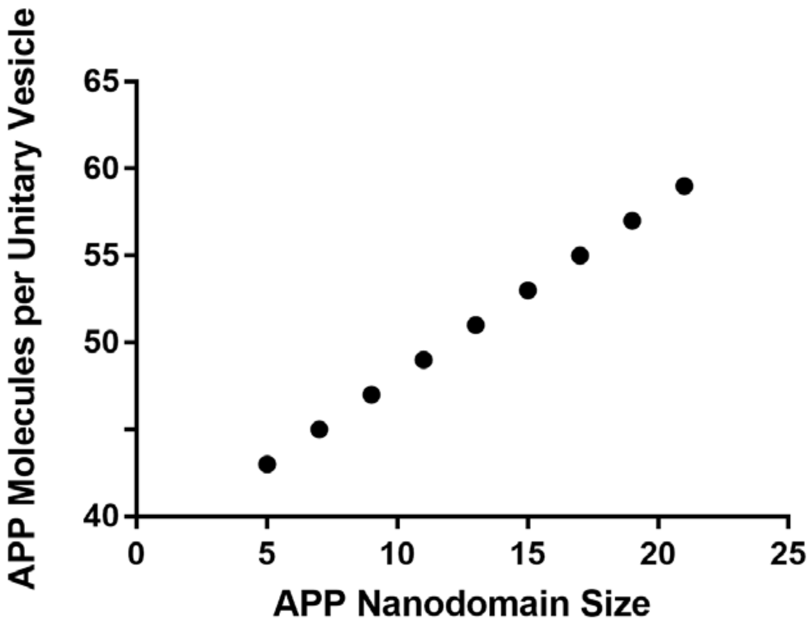

Figure S12. Instantaneous distribution of the density of APP molecules in an endocytic vesicle as a function of APP nanodomain size. Related to Figure 7: Increase in APP cluster size at the endocytic zone results in cumulative increase in APP per unitary endocytic vesicle.

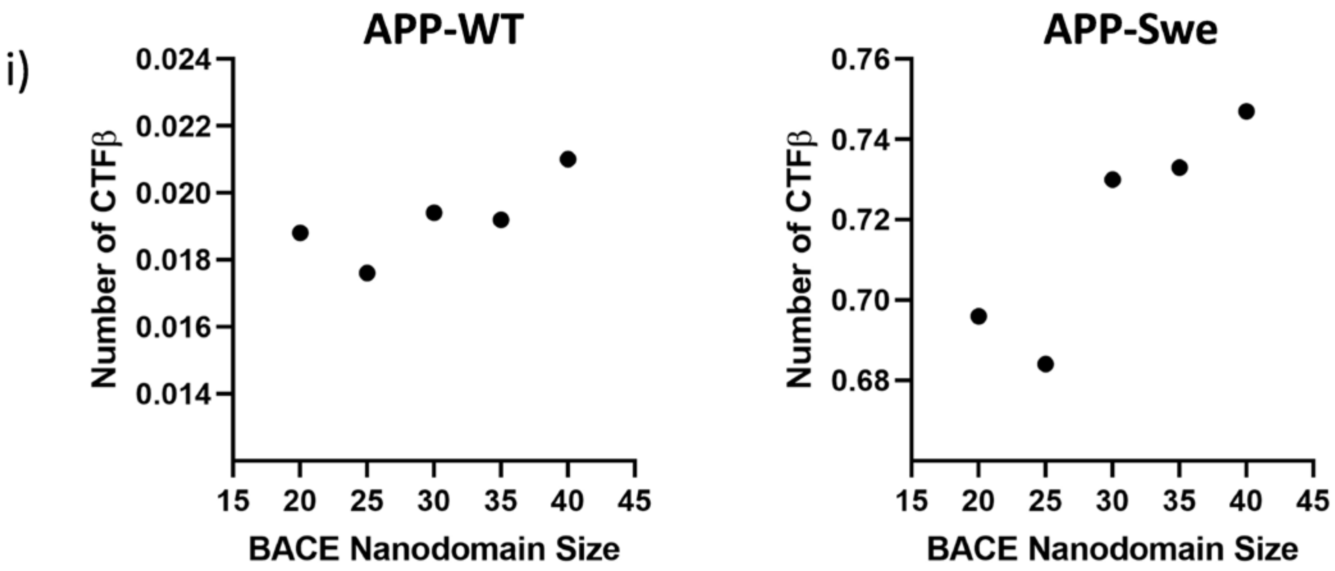

Figure S13. β-secretase clustering increases the probability of production of CTFβ. Related to Figure 7: (i) Probability to produce CTFβ is correlated with the β-secretase nanodomain size internalized per unitary vesicle for APP-WT (left). The results obtained for APP-Swe is indicated in right.

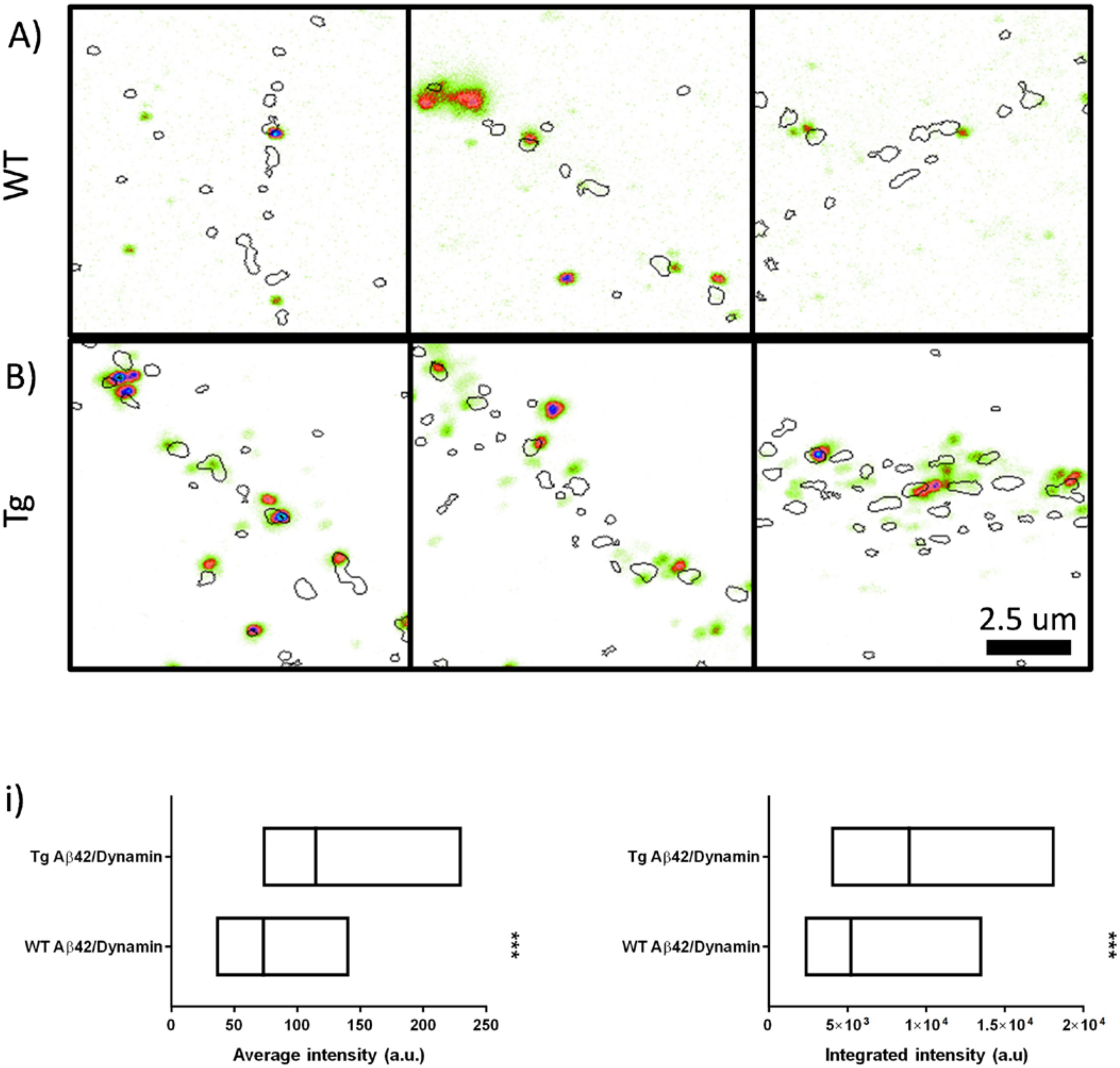

Figure S14. Visualization of Aβ42 in Dynamin enriched regions using confocal microscopy. Related to Figure 7: (A, B) Distribution of Aβ42 in Dynamin enriched regions using confocal microscopy in primary hippocampal neurons obtained from wild type (WT) (A) and APP/PS1 transgenic (Tg) (B) mice. The intensity of Aβ42 is pseudocolour coded from white (minimum) to black (maximum) with black contours representing Dynamin enriched regions. Scale bar at (B) indicate 2.5 µm. (i) Diversity in Aβ42 (median/IQR 25% to 75% interval) average and integrated intensity in Dynamin enriched regions for neurons obtained from WT and APP/PS1 Tg mice. n= 613 (WT Aβ42/Dynamin) and 1068 (Tg Aβ42/Dynamin). Significance was determined by unpaired two-tailed Mann–Whitney test. $^{*}P \leq 0.05$, $^{**}P \leq 0.01$, and $^{***}P \leq 0.001$, ns $P > 0.05$.

Supplementary Tables

Table S1: Summary of quantitative estimation of morphological and biophysical properties of different nanodomains for mouse and human brain slices imaged by STED microscopy (Related to Figure 4, 5 and 6)

Category/Parameter	Length (µm)	Intensity (a.u.)
**WT nanodomain$_{β/PSD}$***	0.152+/-0.003 (0.133, 0.079-0.192)	167.10+/-7.57 (107.90, 53.24-216.70)
**Tg nanodomain$_{β/PSD}$***	0.137+/-0.003 (0.116, 0.083-0.162)	168.90+/-5.44 (118.60, 76.70-206.00)
**WT nanodomain$_{β/EZ}$***	0.113+/-0.004 (0.101, 0.066-0.147)	60.63+/-5.08 (34.57, 16.27-71.54)
**Tg nanodomain$_{β/EZ}$***	0.092+/-0.003 (0.072, 0.033-0.120)	127.90+/-8.74 (82.55, 37.33-146.60)
**Control nanodomain$_{APP/PSD}$****	0.055+/-0.003 (0.046, 0.034-0.068)	18.27+/-6.16 (5.97, 4.96-8.43)
**AD nanodomain$_{APP/PSD}$****	0.074+/-0.004 (0.063, 0.041-0.092)	17.52+/-2.73 (10.22, 8.62-14.47)
**Control nanodomain$_{APP/EZ}$****	0.094+/-0.001 (0.070, 0.044-0.116)	13.32+/-1.03 (7.65, 5.77-11.09)
**AD nanodomain$_{APP/EZ}$****	0.068+/-0.003 (0.053, 0.028-0.083)	96.13+/-13.06 (27.43, 11.95-95.32)
**Control nanodomain$_{β/PSD}$****	0.069+/-0.002 (0.051, 0.034-0.081)	20.42+/-3.68 (7.79, 6.19-10.83)
**AD nanodomain$_{β/PSD}$****	0.070+/-0.003 (0.051, 0.030-0.089)	37.82+/-4.50 (8.38, 6.27-13.72)
**Control nanodomain$_{β/EZ}$****	0.086+/-0.002 (0.067, 0.046-0.112)	24.11+/-3.57 (11.03, 8.77-14.98)
**AD nanodomain$_{β/EZ}$****	0.107+/-0.002 (0.076, 0.047-0.136)	27.88+/-2.73 (14.63, 11.13-21.83)

*Mice slices and **human brain slices. Values indicated are Mean+/-SEM while values in brackets represent median, IQR from 25% percentile to 75% percentile

Table S2: Summary of parameters employed to instantiate the unitary vesicle (Related to Figure 7)

**Parameter**	**Value**
Diameter of Unitary Vesicle*	0.120 µm
Area of Unitary Vesicle*	0.045 μm^2
Density of APP molecules ¶	1000 molecules/μm^2
Density of β-secretase molecules	5600 molecules/μm^2
Number of APP molecules¶	45
Number of β-secretase molecules	252
Number of APP clusters¶	1
Number of β-secretase clusters	0
Area of APP nanodomain¶	0.0056 μm^2
Area of β-secretase nanodomain	No nanodomain used
Diffusion coefficient of APP-WT monomers¶	0.083 μm^2/S
Diffusion coefficient of immobilized APP-WT molecules¶	0.013 μm^2/S
Diffusion coefficient of APP-Swe monomers¶	0.020 μm^2/S
Diffusion coefficient of immobilized APP-Swe molecules¶	0.002 μm^2/S
K_1 APP-WT	4.0×10^{-6} $\mu m^2 Number^{-1} s^{-1}$
K_{-1} APP-WT	0.1 s^{-1}
K_2 APP-WT**	0.002 s^{-1}
K_1 APP-Swe	1.25×10^{-5} $\mu m^2 Number^{-1} s^{-1}$
K_{-1} APP-Swe	0.1 s^{-1}
K_2 APP-Swe**	0.025 s^{-1}
Real-time duration of simulation	5 seconds

**(Ben Halima et al., 2016), *(Kumari et al., 2010; Watanabe et al., 2014), ¶(Kedia et al., 2020)

Table S3: Summary of primary and secondary antibodies used for different experiments (Related to Figure 1, 2, 3, 4, 5 and 6)

Antibody/Experiment	Dilution	Catalogue Number	Company
**Immunocytochemistry (Figures 1, 2, 3 & S1, S3, S14)**			
Anti-PSD95	1:500	MA1-046	Thermo Scientific
Anti-Dynamin	1:1000	05-319	Upstate/Millipore
Anti-APP-CT	1:500	171610	Calbiochem/Millipore
Anti-APP-CT	1:500	MAB343-C	Merck Millipore
Anti-Presenilin1	1:500	MAB5232	Merck Millipore
Anti-Presenilin1	1:500	PRS4203	Sigma-Aldrich
Anti-BACE1*	1:500	840101	Biolegend/Covance
Anti-β-Amyloid (1-42)	1:50	805509	Biolegend/Covance
Alexa Fluor 594	1:400	A11037	Life Technologies
Alexa Fluor 594	1:400	A11032	Life Technologies
Abberior Star Red	1:400	2-0002-011-2	Abberior
**Immunohistochemistry (Figures 4, 5, 6 & S8, S9)**			
Anti-Shank2	1:500	162204	Synaptic Systems
Anti-Clathrin	1:100	ab2731	Abcam
Anti-APP-NT*	1:200	3207	Inhouse Developed
Anti-BACE1*	1:200	840101	Biolegend/Covance
Alexa Fluor 594	1:200	A11037	Life Technologies
Alexa Fluor 488	1:200	A11029	Life Technologies
Alexa Fluor 555	1:200	A21435	Life Technologies
Alexa Fluor 647	1:200	A21245	Life Technologies
Abberior Star Red	1:200	2-0002-011-2	Abberior
Abberior Star Red	1:200	2-0112-011-8	Abberior
**Immunoblotting (Figures 4 & S6)**			
6E10 antibody (1-16)	1:1000	803004	Biolegend/Covance
Anti-BACE	1:500	5606S	Cell Signaling Technology
Anti-APP-CT (CT15)**	1:250	CT15	Inhouse Developed
Anti-β-Actin	1:5000	AC-15 (A1978)	Sigma

*Specificity of the BACE1 antibody (840101) and APP-NT antibody (3207) was in-house validated
**(Wahle et al., 2006)

Table S4: Summary of neuropathological and clinical data for control and AD human case (Related to Figure 5 and 6)

	Age	Gender	PMI	Braak Stage	Aβ Phase	Clinical Diagnosis	Cause of Death
R493/16 (Control)*	92 yrs.	Female	15 h	B2-Braak stage III/IV	A2-Thal phase III	No history of cognitive decline/dementia	Cardiorespiratory arrest
15/B271 (AD)*	92 yrs.	Female	5 h	B2-Braak stage III/IV	A2-Thal phase III	Alzheimer's disease	Pneumonia
12/B243Z (AD)**	82 yrs.	Female	Not available	B1-Braak stage I	A1-Thal phase I	Alzheimer's disease	Pneumonia
19/B352Q (AD)**	76 yrs.	Female	8 hrs	B2-Braak stage III/IV	A2-Thal phase III	Alzheimer's disease	Myocardial infarction
14/B258T (AD)**	83 yrs.	Male	Not available	B2-Braak stage IV	A2-Thal phase III	Alzheimer's disease	Cardiorespiratory arrest
8L2L (Control)**	88 yrs.	Male	Not available	B0	A0	No history of cognitive decline/dementia	Pneumonia
4L2K (Control)**	81 yrs.	Female	Not available	B2-Braak stage III	A2-Thal phase II	No history of cognitive decline/dementia	Cardiorespiratory arrest
9L2R (Control)**	75 yrs.	Male	Not available	B1-Braak stage I	A1-Thal phase I	No history of cognitive decline/dementia	Pneumonia

Post-mortem brain interval time (PMI), *for STED microscopy, **for Airy scan microscopy

Transparent Methods

Experimental Animals

Wild type (WT) C57BL/6 mice or APPswe/PS1ΔE9 double transgenic (Tg) mice (JAX Stock# 004462) were bred at the Institutional Central Animal Facility and were maintained in a temperature-controlled room on 12 h light/12 h dark cycle under pathogen free environment with *ad libitum* access to water and food. All experiments involving animals were carried out in accordance with institutional guidelines for the use and care of animals after approval from the Institutional Animal Ethics Committee (IAEC), Indian Institute of Science, Bangalore, India.

Primary Hippocampal Culture

Mixed sex primary hippocampal neurons cultured from postnatal day 0 or 1 (P0-P1) wild type C57BL/6 mice or APPswe/PS1ΔE9 double transgenic (Tg) mice (JAX Stock# 004462) were prepared and maintained (Kedia et al., 2020). The cells were seeded at a density of 0.1×10^6 cells/mL in 18 mm #1.5 (corrected for 0.17+/- 0.01) glass coverslips (coated with poly-D-lysine at a concentration of 100 µg/mL) in a 12-well cell culture plate. Primary hippocampal neurons were used for immunocytochemical evaluation at DIV 20-21.

Antibodies

The details of the primary and secondary antibodies used in this study are summarized (Table S3).

Immunocytochemistry

Cells were fixed with 4% paraformaldehyde plus 4% sucrose in PBS at 4°C for 10 minutes, followed by quenching with 0.1M glycine in PBS at room temperature and permeabilization with 0.25% Triton X-100 for 5 minutes and then blocked with 10% BSA in PBS for 30 minutes at room temperature. This was followed by incubation with the appropriate primary antibody for 1-2 hr. Following washing, cells were then incubated with a suitable secondary antibody for 45 minutes (Kedia et al., 2020). Following washing, cells were mounted with prolong (Molecular Probes, cat. no. MAN0010261) for confocal or STED imaging.

Mouse Brain Lysates Preparation and Immunoblotting

APPswe/PS1ΔE9 double transgenic (Tg) mice were obtained from Jackson Laboratories, USA (JAX Stock# 004462). The institutional and national guidelines for the care and use of laboratory animals were followed. Brains from 3 months old Tg and or wild type (WT) mice were perfused transcardially with ice-cold saline and removed from the skull. Brain hemispheres were snap-frozen in liquid nitrogen and stored at -80°C until further use. Frozen brains were homogenized with a douncer followed by sonication in 0.32 M sucrose in 50 mM Tris buffer (pH 7.3) containing protease and phosphatase inhibitors (Roche Diagnostics, Germany). Mouse brain homogenates were cleared by centrifugation at 16,100 g and 4°C for 30 minutes. After centrifugation, the resulting supernatant sucrose extract containing the soluble proteins was collected and stored at -80°C (Kumar et al., 2016). The total protein concentration was determined by the BCATM protein assay kit (Thermo Scientific, USA).

Mouse brain lysates were separated by 4–12% NuPAGE (ThermoFisher Scientific) using MES buffer and transferred on to nitrocellulose membranes. The blots were blocked in 3% BSA (diluted in 1x TBS/T) for 2 hours. APP, APP-CTFs, BACE and β-actin Proteins were detected with 6E10 (BioLegend), D10E5 (Cell Signaling Tech) and AC15 (Sigma/Merck) the indicated primary antibodies and respective horseradish peroxidase-conjugated secondary antibodies or conjugated fluorescent antibodies (IRDye 680RD and IRDye 800CW). The same immunoblots were reprobed with anti-β-actin antibody and used as loading control. Immunoreactivity was detected by enhanced chemiluminescence reaction (Biorad) or near infrared detection (LI-COR Odyssey). Band intensities were analyzed using Chemidoc XRS documentation system (Bio-Rad) and Image Studio 5.x CLx software (LI-COR). The bands were compared with an overlay of See Blue prestained ladder (#LC5295, Invitrogen) to compare the bands obtained in the immunoblot.

Immunohistochemistry (Mice)

Mice (3-4 months old) were transcardially perfused with 15 ml of ice-cold PBS followed by 50 ml of ice-cold 4% paraformaldehyde (pH 7.4). The brains were post-fixed in 4% PFA overnight, cryoprotected in 30% sucrose, frozen and stored at -80°C until use. For labelling, 25 µm coronal cryosections from Tg (APPswe/PS1ΔE9, JAX Stock# 004462) mice and control littermates were cut in a cryostat and incubated in blocking/permeabilization solution containing 1% normal goat serum, 3% BSA and 0.3% Triton-X-100 in PBS. Free floating sections were incubated overnight with primary antibodies at 4°C. The sections were washed and incubated with the secondary antibodies at room temperature for 1 h. Following washing, the sections were mounted with prolong with DAPI (Molecular Probes, cat. no. P36962) for confocal/STED imaging. The primary and secondary antibodies used are indicated (Table S3) (Ramanan et al., 2005). The imaging was performed from CA1 to CA2 stratum radiatum layer of the hippocampus.

Human Tissue Collection and Preparation

The brain was removed at autopsy following the written informed consent of next of kin. It was fixed in 10% neutral buffered formalin for 3-4 weeks following which it was sliced serially in the coronal plane. The findings in the gross examination were recorded (cortical atrophy, hippocampal atrophy, infarcts, atherosclerosis). Neuroanatomical areas were sampled in

accordance with the NIAA guidelines for neuropathological assessment for Alzheimer's disease (Hyman et al., 2012). The Ethics Committee at The Human Brain Tissue Repository (Brain Bank) at NIMHANS (Bangalore, India) clearance was obtained for the collection, storage and distribution of human brain tissues for neuroscience research.

Immunohistochemistry (Human)

The tissues were processed for routine paraffin embedding. 4 µm thick serial sections were cut and stained with haematoxylin & eosin stains for morphological assessment. Serial sections were collected on positively charged slides for performing immunohistochemistry by indirect immunoperoxidase method, following antigen retrieval by heat and DAB/H_2O_2 as the chromogen to visualize immunolabeling (DAKO Envision Detection System) to detect neurodegenerative changes using specific antibodies to Paired Helical Filaments (PHF), phosphorylated Tau to detect Neurofibrillary Tangles (NFT), neuritic plaques and neuropil threads (Clone Tau 5, mouse monoclonal antibody, 1:50 dilution, BioGenex, USA) and β-amyloid antibody to detect senile plaques and vascular amyloid deposition (Clone 6F/3D, mouse, monoclonal antibody, 1:100 dilution, Leica Biosystems, USA. The number of Tau positive Neurofibrillary tangles (NFTs), Aβ positive Senile Plaques (SPs) and Diffuse Plaques (DPs) were counted in accordance with published studies (Purohit et al., 2011).

The AD neuropathological changes were ranked according to three to three parameters: the Aβ plaque score (Thal et al., 2002), Braak and Braak NFT stage (Braak et al., 2006) and the CERAD NP score (Mirra et al., 1991) to obtain an "ABC score" reported as 4 levels: not, low, intermediate low, intermediate and high (Montine et al., 2012). The score of the blessed dementia rating scale of the control case was 1.5/17. The summary of staging and pathological details is available (Table S4).

Immunofluorescence (Human)

Paraffin embedded tissue sections were obtained from control and AD cases. Sections were first deparaffinized in xylene and then dehydrated through a series of grades of ethanol. Following quenching and washing, heat induced antigen retrieval was performed using sodium citrate buffer (pH 6.0). Sections were then blocked using universal blocking reagent (cat. no. HK085-5K, BioGenex, USA) for 1 h. Sections were further incubated overnight with primary antibodies at 4°C. Following washing and incubation with the secondary antibodies at room temperature for 1 h and washing, sections were mounted with prolong with DAPI (Molecular Probes, cat. no. P36962) for confocal/STED imaging. Slides were stored at 4°C in dark. The primary and secondary antibodies used are indicated (Table S3). The imaging was performed from CA1 to CA2 radiatum layer of the hippocampus.

Stimulated Emission Depletion microscopy (STED)

A commercial STED inverted microscope (Abberior Expert Line 775 nm, Abberior Instruments GmbH, Göttingen, Germany) was used to obtain confocal and super-resolved images of the same region with a sampling of 15 nm. The microscope was equipped with two pulsed excitation lasers at 561 nm and 640 nm and a pulsed depletion laser at 775 nm. The laser powers were adjusted to 70%, 50% and 40% of their respective total power for 561 nm, 640 nm and 775 nm, respectively (Kedia et al., 2020).

Confocal Microscopy

Confocal microscopy imaging was performed on Zeiss LSM 780 or Zeiss LSM Zeiss 880 at a sampling of 35-100nm/pixel. For each experimental conditions the acquisition and illumination criteria were kept constant (Kedia et al., 2020; Venkatesan et al., 2020).

Airyscan super-resolution Microscopy

Immunohistochemical samples from AD or control human brain co-labelled with nuclear, post synaptic, and endocytic markers together with either APP or BACE were used for super-resolution imaging using Airyscan microscopy. Airyscan was performed on Zeiss LSM 880 equipped with 32 array detectors for acquisition of super-resolution images. We first performed confocal imaging of hippocampal regions using a 40X objective of numerical aperture of 1.3 at a sampling of 11 µm/pixel. Each tile was 350X350 µm^2. We then created a mosaic from the individual tiles to reconstruct the hippocampal region. This was performed by tiling images to generate a mosaic image of 3.5X3.5 mm^2 to 4.5X4.5 mm^2. We then performed Airyscan imaging on 5-10 non-overlapping regions from the radiatum layer of the hippocampus with an effective field size of 75 X75 µm^2 sampled at 35 nm/pixel using a 63X objective with a numerical aperture of 1.4. For image acquisition, 405, 488, 543 and 633 nm lasers were used. The illumination intensities, sampling of the images, digital and analogue gain of the detectors, emission window for each fluorescent channel and their corresponding pinhole sizes were maintained constant across acquisition. The raw images acquired using Airyscan mode were processed using Zeiss acquisition and analysis software of the microscope to generate final super-resolution images. The reconstruction parameters were kept constant throughout the samples.

Semiautomated detection of dendritic compartments and functional zones of an excitatory synapse

The active dendritic area of protein of interest and synapses were distinguished from the rest of the dendrite using a custom defined segmentation protocol. The intensity of the epifluorescence/confocal images of markers for different functional zones of the synapse (post/peri) was thresholded to generate the mask of the puncta. A spine morphometry analysis was then performed, and masks were filtered using various morphological filters like length, breadth and area through IMA plugin

running inside MetaMorph software (Molecular Devices). Similar analysis was performed on super-resolution images to detect functional zones of an excitatory synapse (post/peri) that correspond to PSD/EZ functional zones (Kedia et al., 2020).

Resolution Scaled Pearson's Coefficient (RSP) and Resolution Scaled Error (RSE) Analysis
RSP and RSE were calculated using NanoJ-SQUIRREL, a plugin supported by Fiji (Culley et al., 2018; Venkatachalapathy et al., 2019). The reference image was either a marker for functional zones of the synapse for β/γ-secretase on post/peri analysis or APP or γ-secretase for β/γ-secretase on APP and β-secretase on γ-secretase analysis (Kedia et al., 2020). The subject image was of the protein of interest (secretases). Both reference and subject images were super-resolution images.

Super-resolution Cluster Analysis
Clusters of molecular aggregation (nanodomains) were identified from super-resolution images by a custom algorithm written as a plug-in supported by MetaMorph (Molecular Devices) (Izeddin et al., 2012; Kechkar et al., 2013; Nair et al., 2013). Nanodomains were detected from super-resolution images using the Palm-Tracer plugin. Nanodomains were analyzed using bi-dimensional Gaussian fitting, from which the principal ($2.3\sigma_{long}$) and the auxiliary axes ($2.3\sigma_{short}$, data not shown) were determined for STED while for the data from Airyscan, length$_{\sigma}$ (σ_{long}) was plotted. The fitting was performed on each cluster that was identified as a domain. Several parameters like area and intensity of nanodomain were computed for each experimental category. The intensity of the nanodomain was normalized with the global median/mean value to allow relative comparison of the content of nanodomains either between different pools of nanodomains when associated with functional zones of the synapse or with other components of amyloidogenic machinery.

Nearest Neighbor Distance Measurement
The nearest neighbor distances (NND) were calculated using interaction analysis, a plugin supported by Fiji (Helmuth et al., 2010; Kedia et al., 2020; Shivanandan et al., 2013). The reference image (Y) was post/perisynaptic marker and image (X) was the protein of interest in all the categories where analysis is performed between the synaptic marker and the protein of interest. While for BACE-APP, PS-APP and BACE-PS, the NNDs were calculated with Y as APP for BACE-APP, PS-APP and as PS for BACE-PS. The ROUT method of identifying outliers with Q= 1% was used for NND measurements.

Model Components and Geometry
Simulations were performed in a spherical vesicle of diameter 120 nm to reflect a typical endocytic vesicle. The details of variables used to instantiate the unitary vesicle is summarized in Table S2. MCell, *version* 3, a Monte Carlo Cell simulator, was used to carry out the simulations. Monte Carlo algorithms were used by MCell to simulate the diffusion of individual molecules present either on a surface or in a confined volume (Kerr et al., 2008). MCell carries out user-specified molecular reactions stochastically. These simulations track each molecule and the relevant reactions to calculate spatiotemporal trajectories. Simulations were performed on a cluster with 1464 processing units. Several thousands of trajectories (1000-5000, the higher number of trajectories simulated for reactions with low reaction rates for greater confidence) were simulated to compute the average reaction diffusion trajectory for APP with β-secretase.

Model Configurations
Amyloidogenic processing of APP occurs due to sequential activities of β- and γ-secretases. Processing by β-secretase is a critical and necessary condition for the generation of Aβ. To understand this processing step, β-secretase and APP were modelled with varying compositionality inside endocytic vesicles. The origin of these endocytic vesicles was randomly chosen from perisynaptic compartments, where the distribution of these molecules was quantified through nanoscopy. The following three assumptions were then used to define the characteristics of endocytic vesicles. 1) Endocytic vesicles originate from EZ and are instantiated as confined spherical membrane bound organelles with a luminal pH of 5.5 and a diameter of 20 nm. 2) β-secretase is distributed uniformly in the vesicular membrane while the compositionality of APP changes from a vesicle is populated with 36 single molecules of APP with a combination of no nanodomains or nanodomain$_{APP}$ of 5, 9 or 13 confined APP molecules residing in an area of 0.0056 μm^2. 3) The vesicle has no other substrates for β-secretase apart from APP. Approximately 250 β-secretase molecules were distributed uniformly on the vesicles. The diffusion coefficient of the confined APP molecules within nanodomain$_{APP}$ (Table S2) was taken to be 1×10^{-2} $\mu m^2/s$, while that of APP monomer was taken to be 7×10^{-2} $\mu m^2/s$ (Kedia et al., 2020). Since β-secretase is a single pass transmembrane protein like APP and its diffusion coefficient has been estimated to be of the same order of magnitude as the APP monomer, we assumed the diffusion coefficient of β-secretase monomer to be 7×10^{-2} $\mu m^2/s$ as well. To simulate APP- β-secretase reaction, we assumed the APP processing to follow Michaelis- Menten kinetics (Ben Halima et al., 2016). Both the rates k_{cat} (catalytic rate) and the k_M (the Michaelis constant) for APP-WT and APP-Swe have been measured by Ben Halima et. al and were used here for the simulations (Ben Halima et al., 2016). As these constants were obtained experimentally under conditions that facilitated 3D diffusion of molecules, only the k_{cat} value was taken from their data while the k_1 and k_{-1} were set to values such that a reasonable number of intermediate molecules were formed. k_M for APP-Swe was approximately 7 times that of APP-WT and was simulated appropriately. The k_{cat} value for APP-Swe was used as reported in Ben Halima et al., 2016 (Ben Halima et al., 2016). The details of variables used for the simulation is summarized (Table S2).

Statistics
Statistical analysis and significances were performed using GraphPad Prism version 7.04 for Windows, GraphPad Software, La Jolla California USA, (www.graphpad.com). D'Agostino-Pearson Omnibus normality test and Shapiro-Wilk normality test were used to test normal distribution. All statistical values were shown as mean +/- SEM for normally distributed data or median (IQR 25% to 75% interval) for non-normally distributed data, unless otherwise indicated. Tabulated summary data are presented as mean +/- SEM (median, IQR 25% to 75% interval). Normally distributed datasets were compared using two-tailed unpaired Student's t-test (for two-group), one-way analysis of variance (ANOVA) test followed by Tukey's multiple comparison test (for multi-group). Non-normally distributed datasets were tested by non-parametric two-tailed Mann-Whitney test (for two-group) or Kruskal-Wallis test followed by Dunn's multiple comparison test (for multi-group). Indications of significance correspond to P values $*P \leq 0.05$, $**P \leq 0.01$, and $***P \leq 0.001$, ns $P > 0.05$. The calculation of the required sample sizes was obtained from the power and sample size calculator from statistical solutions (Nair et al., 2013). To account for variability, data were obtained from 2-4 independent cultures/animals.

Schemes
All the schemes and graphical abstract were prepared using Biorender.com

References

Ben Halima, S., Mishra, S., Raja, K.M.P., Willem, M., Baici, A., Simons, K., Brustle, O., Koch, P., Haass, C., Caflisch, A.*, et al.* (2016). Specific Inhibition of beta-Secretase Processing of the Alzheimer Disease Amyloid Precursor Protein. Cell Rep *14*, 2127-2141.
Braak, H., Alafuzoff, I., Arzberger, T., Kretzschmar, H., and Del Tredici, K. (2006). Staging of Alzheimer disease-associated neurofibrillary pathology using paraffin sections and immunocytochemistry. Acta Neuropathol *112*, 389-404.
Culley, S., Albrecht, D., Jacobs, C., Pereira, P.M., Leterrier, C., Mercer, J., and Henriques, R. (2018). Quantitative mapping and minimization of super-resolution optical imaging artifacts. Nat Methods *15*, 263-266.
Helmuth, J.A., Paul, G., and Sbalzarini, I.F. (2010). Beyond co-localization: inferring spatial interactions between sub-cellular structures from microscopy images. BMC Bioinformatics *11*, 372.
Hyman, B.T., Phelps, C.H., Beach, T.G., Bigio, E.H., Cairns, N.J., Carrillo, M.C., Dickson, D.W., Duyckaerts, C., Frosch, M.P., Masliah, E.*, et al.* (2012). National Institute on Aging-Alzheimer's Association guidelines for the neuropathologic assessment of Alzheimer's disease. Alzheimers Dement *8*, 1-13.
Izeddin, I., Boulanger, J., Racine, V., Specht, C.G., Kechkar, A., Nair, D., Triller, A., Choquet, D., Dahan, M., and Sibarita, J.B. (2012). Wavelet analysis for single molecule localization microscopy. Opt Express *20*, 2081-2095.
Kechkar, A., Nair, D., Heilemann, M., Choquet, D., and Sibarita, J.-B. (2013). Real-time analysis and visualization for single-molecule based super-resolution microscopy. PLoS One *8*, e62918.
Kedia, S., Ramakrishna, P., Netrakanti, P.R., Jose, M., Sibarita, J.B., Nadkarni, S., and Nair, D. (2020). Real-time nanoscale organization of amyloid precursor protein. Nanoscale *12*, 8200-8215.
Kerr, R.A., Bartol, T.M., Kaminsky, B., Dittrich, M., Chang, J.C., Baden, S.B., Sejnowski, T.J., and Stiles, J.R. (2008). Fast Monte Carlo Simulation Methods for Biological Reaction-Diffusion Systems in Solution and on Surfaces. SIAM J Sci Comput *30*, 3126.
Kumar, S., Wirths, O., Stuber, K., Wunderlich, P., Koch, P., Theil, S., Rezaei-Ghaleh, N., Zweckstetter, M., Bayer, T.A., Brustle, O.*, et al.* (2016). Phosphorylation of the amyloid beta-peptide at Ser26 stabilizes oligomeric assembly and increases neurotoxicity. Acta Neuropathol *131*, 525-537.
Kumari, S., Mg, S., and Mayor, S. (2010). Endocytosis unplugged: multiple ways to enter the cell. Cell Res *20*, 256-275.
Mirra, S.S., Heyman, A., McKeel, D., Sumi, S.M., Crain, B.J., Brownlee, L.M., Vogel, F.S., Hughes, J.P., van Belle, G., and Berg, L. (1991). The Consortium to Establish a Registry for Alzheimer's Disease (CERAD). Part II. Standardization of the neuropathologic assessment of Alzheimer's disease. Neurology *41*, 479-486.
Montine, T.J., Phelps, C.H., Beach, T.G., Bigio, E.H., Cairns, N.J., Dickson, D.W., Duyckaerts, C., Frosch, M.P., Masliah, E., Mirra, S.S.*, et al.* (2012). National Institute on Aging-Alzheimer's Association guidelines for the neuropathologic assessment of Alzheimer's disease: a practical approach. Acta Neuropathol *123*, 1-11.
Nair, D., Hosy, E., Petersen, J.D., Constals, A., Giannone, G., Choquet, D., and Sibarita, J.B. (2013). Super-resolution imaging reveals that AMPA receptors inside synapses are dynamically organized in nanodomains regulated by PSD95. J Neurosci *33*, 13204-13224.
Purohit, D.P., Batheja, N.O., Sano, M., Jashnani, K.D., Kalaria, R.N., Karunamurthy, A., Kaur, S., Shenoy, A.S., Van Dyk, K., Schmeidler, J.*, et al.* (2011). Profiles of Alzheimer's disease-related pathology in an aging urban population sample in India. J Alzheimers Dis *24*, 187-196.
Ramanan, N., Shen, Y., Sarsfield, S., Lemberger, T., Schutz, G., Linden, D.J., and Ginty, D.D. (2005). SRF mediates activity-induced gene expression and synaptic plasticity but not neuronal viability. Nat Neurosci *8*, 759-767.
Shivanandan, A., Radenovic, A., and Sbalzarini, I.F. (2013). MosaicIA: an ImageJ/Fiji plugin for spatial pattern and interaction analysis. BMC Bioinformatics *14*, 349.
Thal, D.R., Rub, U., Orantes, M., and Braak, H. (2002). Phases of A beta-deposition in the human brain and its relevance for the development of AD. Neurology *58*, 1791-1800.

Venkatachalapathy, M., Belapurkar, V., Jose, M., Gautier, A., and Nair, D. (2019). Live cell super resolution imaging by radial fluctuations using fluorogen binding tags. Nanoscale.

Venkatesan, S., Subramaniam, S., Rajeev, P., Chopra, Y., Jose, M., and Nair, D. (2020). Differential Scaling of Synaptic Molecules within Functional Zones of an Excitatory Synapse during Homeostatic Plasticity. eNeuro *7*.

Wahle, T., Thal, D.R., Sastre, M., Rentmeister, A., Bogdanovic, N., Famulok, M., Heneka, M.T., and Walter, J. (2006). GGA1 is expressed in the human brain and affects the generation of amyloid beta-peptide. J Neurosci *26*, 12838-12846.

Watanabe, S., Trimbuch, T., Camacho-Perez, M., Rost, B.R., Brokowski, B., Sohl-Kielczynski, B., Felies, A., Davis, M.W., Rosenmund, C., and Jorgensen, E.M. (2014). Clathrin regenerates synaptic vesicles from endosomes. Nature *515*, 228-233.
